# Small nucleolar RNA *SNORA71A* promotes epithelial‐mesenchymal transition by maintaining *ROCK2* mRNA stability in breast cancer

**DOI:** 10.1002/1878-0261.13186

**Published:** 2022-03-30

**Authors:** Ting Hu, Chong Lu, Yun Xia, Lu Wu, Junlong Song, Chuang Chen, Qiong Wang

**Affiliations:** ^1^ 12443 Cancer Center Union Hospital Tongji Medical College Huazhong University of Science and Technology Wuhan China; ^2^ 12443 Department of Thyroid and Breast Surgery Union Hospital Tongji Medical College Huazhong University of Science and Technology Wuhan China; ^3^ 117921 Department of Breast and Thyroid Surgery Renmin Hospital of Wuhan University China

**Keywords:** breast cancer, epithelial‐mesenchymal transition, *ROCK2*, *SNORA71A*, snoRNA

## Abstract

Metastasis is the primary reason of death in patients with cancer. Small nucleolar noncoding RNAs (snoRNAs) are conserved 60–300 nucleotide noncoding RNAs, involved in post‐transcriptional regulation of mRNAs and noncoding RNAs. Despite their essential roles in cancer, the roles of snoRNAs in epithelial‐mesenchymal transition (EMT)‐induced metastasis have not been studied extensively. Here, we used small RNA sequencing to screen for snoRNAs related to EMT and breast cancer metastasis. We found a higher expression of *SNORA71A* in metastatic breast cancer tissues compared to nonmetastatic samples. Additionally, *SNORA71A* promoted the proliferation, migration, invasion and EMT of MCF‐7 and MDA‐MB‐231 cells. Mechanistically, *SNORA71A* elevated mRNA and protein levels of *ROCK2*, a negative regulator of TGF‐β signaling. Rescue assays showed *ROCK2* abrogated the *SNORA71A*‐mediated increase in proliferation, migration, invasion and EMT. Binding of *SNORA71A* to mRNA stability regulatory protein G3BP1, increased *ROCK2* mRNA half‐life. Furthermore, G3BP1 depletion abolished the *SNORA71A*‐mediated upregulation of *ROCK2*. *In vivo*, *SNORA71A* overexpression promoted breast tumor growth, and *SNORA71A* knockdown inhibited breast cancer growth and metastasis. We suggest *SNORA71A* enhances metastasis of breast cancer by binding to G3BP1 and stabilizing *ROCK2*.

## Graphical abstract

The role of snoRNAs in cancer metastasis has not been studied in detail. We observed that *SNORA71A* promoted metastasis of breast cancer cells *in vitro* and *in vivo*. Mechanically, *SNORA71A* recruited G3BP1 to the mRNA of *ROCK2*, thus increasing the mRNA stability of *ROCK2*, and further promoting EMT via TGF‐β signaling.

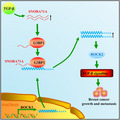

**Abbreviations**
CCK‐8: Cell Counting Kit‐8
DMEM: Dulbecco's modified Eagle's medium
EdU: ethynyldeoxyuridine
EMT: epithelial‐to‐mesenchymal transition
GO: Gene Ontology
IgG: anti‐immunoglobulin G
KEGG: Kyoto Encyclopedia of Genes and Genomes
MS: mass spectrometry
NC: negative control
nt: nucleotides
RIP: RNA immunoprecipitation
ROC: receiver operating characteristic
SMA: smooth muscle actin
snoRNAs: small nucleolar noncoding RNAs
TGF‐β: transforming growth factor

## 1 Introduction

Breast cancer is one of the most common carcinomas in women worldwide. In 2018, approximately 2.1 million breast cancer cases were diagnosed and caused 626 679 deaths [1]. The mean age of diagnosis for breast cancer is 62, and it is estimated that one out of eight females might develop breast cancer at some point in their lives [2]. Metastatic breast cancer needs to be treated according to the subtypes to prolong life and reduce symptoms [3]. Although significant advances have been made in the treatment of breast cancer, the prognosis of patients with metastasis is still poor. The median overall survival for metastatic triple‐negative breast cancer and the other two subtypes (hormone receptor positive and ERBB2 positive) are about 1 and 5 years, respectively [3]. Development of cancer metastasis implicates various mechanisms, including angiogenesis, migration, invasion, and epithelial‐mesenchymal transition (EMT) [4]. Therefore, there is an urgent need to investigate the underlying mechanisms of EMT to develop more accurate prognostic markers and effective therapeutic strategies.

Epithelial‐to‐mesenchymal transition indicates the phenotypic conversion of epithelial cells to mesenchymal cells, which is pivotal for invasion and metastasis of cancer cells [5]. This alternation in cell behavior is mediated by key transcription factors, among which transforming growth factor (TGF‐β) signaling plays a predominant role [6]. TGF‐β induces the phosphorylation activity of SMAD 2/3 to mediate the regulation of target genes on the transcription level, thus promoting EMT, metastasis and tumorigenesis [7, 8]. Activation of EMT ultimately leads to the loss of epithelial markers, including E‐cadherin and cytokeratin, as well as to an increase of mesenchymal markers such as Vimentin, smooth muscle actin (SMA), and matrix‐degrading enzymes.

Small nucleolar noncoding RNAs (snoRNAs) are a group of conserved noncoding RNAs with the length of 60–300 nucleotides (nt). They are widely distributed in the eukaryotic cell nucleolus and are primarily divided into box C/D snoRNAs and box H/ACA snoRNAs [9]. snoRNAs direct the demethylation and pseudo‐uracylation of ribosomal RNA through base‐pairing and are involved in post‐transcriptional modification of snRNAs, tRNAs, and mRNAs [10, 11]. snoRNAs have been shown to play essential roles in cancer. For instance, *SCARNA13* facilitates metastasis of hepatocellular carcinoma by regulating SOX9 [12]. *SNORA71A* increases migratory and invasive capacity in lung carcinoma by mediating the MEK and ERK1/2 phosphorylation levels [13]. H/ACA box small nucleolar RNA 7B has been shown to promote breast cancer [14, 15]. However, the role of snoRNAs in EMT progression of breast cancer has not been studied extensively.

Here, we aimed to identify and evaluate the role of snoRNAs as potentially suitable therapeutic targets for TGF‐β‐mediated EMT in breast cancer. We explored EMT‐related snoRNAs by small RNA sequencing in TGF‐β‐stimulated breast cancer and the control cells. The function of a key snoRNA in EMT was verified by CCK‐8, transwell, and western blot analyses, and the mechanisms were investigated by RNA sequencing, real‐time PCR, and western blotting.

## 2 Materials and methods

### 2.1 Ethics approval and consent to participate

The present study was approved by the ethical review committees of China. Ethical approval was granted from the Ethical Review Committees of Tongji Medical College, Huazhong University of Science and Technology. This study was performed in accordance with the Declaration of Helsinki. Written informed consent was obtained from all the patients. The animal experiments in this study and all procedures involving the handling and treatment of mice during this study were approved by the Ethical Review Committees of Tongji Medical College, Huazhong University of Science and Technology. All the experiments were performed according to the guidelines of the National Institutes of Health Guide for the Care and Use of Laboratory Animals.

### 2.2 Human tissues

Breast cancer tissues and adjacent healthy tissues were obtained following curative surgical resections from 39 patients with breast cancer at the hospital. The patient information is shown in Table S1.

### 2.3 Cell culture and TGF‐β treatment

The human cell lines of breast cancer, including MCF‐7 and MDA‐MB‐231, were purchased from Procell (Wuhan, China). MCF‐7 cells were cultured in Dulbecco's modified Eagle's medium (DMEM, 10‐013‐CVR; Corning, Manassas, VA, USA) with 10% (v/v) heat‐inactivated FBS (GIBCO, Grand Island, NY, USA) and 1% penicillin‐streptomycin (Sangon, Shanghai, China), in a humidified incubator with 5% CO_2_ at 37 °C. MDA‐MB‐231 cells were incubated in Leibovitz's 15 medium (L15, E600016‐0500; BBI Life Sciences, Shanghai, China) with 10% (v/v) FBS and 1% P/S, in an incubator with 5% CO_2_ at 37 °C. Cells were induced into the EMT model by treating with 20 ng·mL^−1^ TGF‐β1 (100‐21‐10; PeproTech, Cranbury, NJ, USA) for 48 h.

### 2.4 Plasmid construction and cell transfection

The siRNA fragments targeting *SNORA71A* were synthesized in Gene Pharma (Shanghai, China), and the scramble siRNA was used as a negative control (NC). *SNORA71A* was overexpressed by cloning its whole length sequence into pLVX‐EGFP‐IRES‐Puro vector, using EcoRI (GAATTC) and XbaI (TCTAGA) cloning sites. Overexpressing plasmids of *ROCK2* were obtained by amplifying their complete sequences and cloning into the overexpression vector pcDNA 3.1. For cell transfection, cells were seeded in 6‐well plates with a density of 3 × 10^5^ cells/well till confluence of 80–90% after 24 h. Next, cells were cultured with fresh medium and transfected with siRNA fragments or overexpressing plasmids using LipofectamineTM2000 (Invitrogen, Carlsbad, CA, USA). The siRNA sequences are shown in Table S2.

### 2.5 RNA isolation

Total RNA of cells and tissues was extracted via the TRIzol method (Invitrogen). The quality and quantity of total RNA was detected by NanoDrop ND1000 Spectrophotometer (Thermo Scientific, Waltham, DE, USA). RNA samples with A260/280 > 1.9 were used for real‐time PCR and cDNA library construction.

### 2.6 snoRNA library preparation and sequencing

TGF‐β‐treated MCF‐7, MDA‐MB‐231, and the corresponding control cells were subjected for snoRNA sequencing. snoRNA libraries were constructed by NEB Small RNA Library Prep kit (cat. no. E7560S; New England Biolabs, Inc., Ipswich, MA, USA). Briefly, total RNA was ligated with 3′‐ and 5′‐adapters, and cDNA was synthesized via PCR. The DNA fragments with 180–420 bp (including 120 bp adapters) were extracted by the QIAquick gel extraction kit (Qiagen, Valencia, CA, USA). The four libraries were sequenced on an Illumina HiSeq 2500 (Illumina, San Diego, CA, USA) in Ying Biotech (http://www.yingbio.com).

### 2.7 mRNA library construction and sequencing

mRNA sequencing was operated in *SNORA71A*‐overexpressing MDA‐MB‐231 and negative control cells. Libraries were constructed via VAHTS™ Total RNA‐seq (H/M/R) Library Prep Kit (#NR603; Vazyme Biotech, Nanjing, China) following the user guides. In brief, mRNA was extracted using oligo‐dT magnetic beads and processed to mRNA fragments under divalent cations and high‐temperature conditions. Fist‐strand cDNA was synthesized by reverse transcriptase using random primers. The cDNA libraries were constructed via PCR and purified using Ampure Beads (Beckman, Brea, CA, USA). The quality of the DNA fragment was measured by Agilent 2200 (Agilent, Santa Rosa, CA, USA). Finally, RNA sequencing was performed using an Illumina HiSeq 2500 platform.

### 2.8 Sequencing data analysis

Short reads (< 15 nt), adaptor sequences, and low‐quality sequences (> 50% of bases whose *Q* scores were ≤ 10%) were removed from the raw sequencing data using fast‐qc (v0.11.7) (http://www.bioinformatics.babraham.ac.uk/projects/fastqc/). The clean data were mapped to the human genomic reference (GRCH38 version), and the snoRNA was identified by mapping to the snoRNA database (RNAcentral V18). The differentially expressed snoRNA between TGF‐β group and control group was analyzed by DEGseq in MCF‐7 and MDA‐MB‐231, respectively, and the |fold change| > 1.5, FDR < 0.05 was consider significant. The differentially expressed mRNAs were identified based on |fold change| > 2, FDR < 0.05 selection. Functions of the different genes were classified by Gene Ontology (GO) and Kyoto Encyclopedia of Genes and Genomes (KEGG) database. Heatmaps were performed using differently expressed genes. GSEA was performed by using all identified genes (including genes that did not differ significantly) for enrichment analysis.

### 2.9 Analysis of expression data from database

The expression data of snoRNA from 1077 breast cancer samples and 105 normal samples were downloaded from The Cancer Genome Atlas (TCGA) database (http://bioinfo.life.hust.edu.cn/SNORic/download/). Among the 1078 cancer samples, 1076 had clinical information, which was used for survival analysis. We used the median score in training set as the cutoff and the data were divided into low‐risk and high‐risk groups. The Kaplan–Meier (KM) and log‐rank methods were used to compare the survival rate between low‐ and high‐risk groups via the r ‘survival’ package (version 3.5.2). All data were publicly available and were downloaded for research purpose.

### 2.10 Cell proliferation assay

Cell Counting Kit‐8 (CCK‐8) assay was used to evaluate cell proliferation. Cells were cultured in the 96‐well plates with 3 × 10^3^ cells/well. Cells were transfected with overexpressing plasmids or siRNA fragments and cultivated for 24, 48, and 72 h, respectively, before the addition of 10 μL CCK‐8 reagent (Dojindo, Kumomoto, Japan) and further incubation for 3 h at 37 °C. The proliferation potential was evaluated by determination of OD value at 450 nm using the microplate reader (Infinite M1000; TECAN, Männedorf, Switzerland).

### 2.11 Cell cycle analysis

Breast cancer cells were starved for 24 h and next cultured in fresh medium containing 10% FBS for 24 h. We then collected the breast cancer cells and fixed them in 75% ethanol under 4 °C, overnight. Cells were suspended and culture in propidium iodide (PI)/RNase staining buffer (BD Biosciences, San Jose, CA, USA) in the dark, for 15 min flow cytometer (BD Biosciences) was used to analyze cell cycle.

### 2.12 Cell migration and invasion assays

Cell migration experiment was performed using 0.8‐μm 24‐well chamber (353097, FALCON, Corning), and invasion assay was performed with BioCoat™ Matrigel^®^ 0.8‐μm 24‐well chamber (354480; Corning). A total of 700 μL medium containing 10% serum was added in the lower chamber, and 500 μL cell suspension was added in the upper chamber and cultured for 24 h. The liquid in the upper chamber was soaked away, and the cells attaching the bottom of the upper chamber were stained using 800 μL crystal violet dye (Sigma, St. Louis, MO, USA) for 30 min at 20 °C. Results were evaluated in three random fields under the microscope.

### 2.13 Ethynyldeoxyuridine analysis

Ethynyldeoxyuridine (EdU) detection kit (RiboBio, Guangzhou, China) was used to assess cell proliferation according to the manufacturer's instruction. Cells were cultured in 96‐well plates at 5 × 10^3^ cells/well. Ten microliters of EdU labeling media was added to the 96‐well plates and then incubated at 37 °C under 5% CO_2_ for 2 h. After treatment with 4% paraformaldehyde (PFA; Sigma) and 0.5% Triton X‐100 (Sigma), the cells were stained with the anti‐EdU working solution and Hoechst 33342 (Sigma). Subsequently, the cells were visualized using a fluorescence microscope (Olympus, Tokyo, Japan). The EdU incorporation rate was calculated as the ratio of the number of EdU‐positive cells (green cells) to the total number of Hoechst 33342‐positive cells (blue cells).

### 2.14 RNA pull‐down assay

The *SNORA71A* probe was synthesized by *in vitro* transcription using complete cDNA sequence of *SNORA71A* as a template, via a T7 *in vitro* Transcription Kit (Thermo Fisher Scientific, Waltham, MA, USA). The antisense of *SNORA71A* was used as a negative control probe. Magnetic RNA‐Protein Pull‐Down Kit (Thermo Fisher Scientific) was used according to the protocol specified by the manufacturer's instructions for RNA pull‐down. MDA‐MB‐231 cell lysates were incubated with purified biotinylated transcripts for 1 h at 25 °C. The biotin‐coupled RNA complexes were isolated by streptavidin agarose beads (Invitrogen). The pellets were washed and then boiled with a loading buffer. Then, the pull‐down material was analyzed.

### 2.15 Mass spectrometry (MS)

The specific protein bands were cut and subjected to washing, decolorization, and dehydration. Samples were supplemented with 10 μL enzymatic hydrolysate for 30 min and 20 μL enzymatic hydrolysate cover solution, and subjected to enzymolysis in water bath at 37 °C for 16 h. The dried polypeptide sample was re‐dissolved in Nano‐HPLC Buffer A, activated by C18 column using 40 μL methanol, and balanced using 40 μL Nano‐HPLC Buffer A, desalted using 40 μL Nano‐HPLC Buffer A, and washed with 40 μL Nano‐HPLC Buffer B. The enzymatic hydrolysis products were separated by High‐Performance Liquid Chromatography by Agilent liquid chromatograph and analyzed via mass spectrometry by Q‐Exactive HF mass spectrometer (Thermo Scientific). The data were processed via proteome discoverer software (version 2.1; Thermo Fisher).

### 2.16 RNA immunoprecipitation assay

The Magna RIP™ RNA‐Binding Protein Immunoprecipitation Kit (Millipore, Bedford, MA, USA) was used according to the protocol specified for RNA immunoprecipitation (RIP) assay. Complete RIP lysis buffer was used to lyse MDA‐MB‐231 cells, which were then centrifuged at 16 000 *g* for 10 min. Cell lysates were incubated with the magnetic beads conjugated with anti‐G3BP1 (1 : 20, # ab181150; Abcam, Cambridge, UK) or control anti‐immunoglobulin G (IgG) antibody. Beads were washed three times with RIP buffer and once with PBS buffer. The immunoprecipitated RNA product was purified and subjected to quantitative real‐time PCR.

### 2.17 Actinomycin D experiment

Actinomycin D was used to examine the effect of *SNORA71A* on the stability of *ROCK2* mRNA. In brief, Actinomycin D (5 μg·mL^−1^, A4262; Sigma) was added to MDA‐MB‐231 cells, and the real‐time PCR was performed at 0, 3, 6, 9, and 12 h after Actinomycin D treatment.

### 2.18 Real‐time PCR

Complementary DNA was synthesized from the RNA using a reverse transcription kit (Thermo Bio, Waltham, MA, USA). PCR was performed using 2 × Master Mix kit (Roche, Basel, Switzerland) following the manufacturer's instructions, and assessed on an ABI Q6 (Applied Biosystems Inc., Carlsbad, CA, USA) thermocycler. The amplification program was 95 °C for 10 min, followed by 40 cycles at 95 °C for 15 s and 60 °C for 60 s. The primer sequences are shown in Table S2. The expression levels were calculated using 2^−ΔΔCT^ method. The expression level of GAPDH was used as the reference gene for normalization.

### 2.19 Western blotting

Cells were lysed with RIPA buffers (Thermo Fisher Scientific), and equal amounts of protein for each sample were separated by 10% SDS/PAGE and transferred to the polyvinylidene fluoride membrane (Sangon Biotech, Shanghai, China). The membranes were incubated with primary antibodies, including E‐cadherin (1 : 1000, Cell Signaling Technology, Boston, MA, USA), Vimentin (1 : 1000; Cell Signaling Technology), ROCK2 (1 : 1000; Cell Signaling Technology), and GAPDH (1 : 1000; Absin Bioscience Inc., Shanghai, China), respectively, at 4 °C overnight. Membranes were washed and incubated with the HRP‐conjugated goat anti‐mouse IgG (1 : 1000; Beyotime, Shanghai, China) or goat anti‐rabbit IgG (1 : 5000; Abcam) for 2 h. The protein bands were detected by ECL (Thermo Fisher Scientific).

### 2.20 Immunofluorescence assay

Cells were seeded on the coverslips and fixed via 4% paraformaldehyde at 20 °C for 20 min. The attached cells were washed three times using PBS and permeabilized by 0.1% Triton X‐100 for 3 min. The coverslips were blocked by blocking buffer for 1 h, and incubated in presence of primary antibodies against E‐cadherin (1 : 1000; Cell Signaling Technology) and Vimentin (1 : 1000; Cell Signaling Technology) for 2 h. Cells were then treated with a fluorescently labeled secondary antibody (Abcam, London, UK) for 30 min. The cell nuclei were observed by staining with DAPI for 5 min. Images were obtained using a fluorescence microscope.

### 2.21 RNA‐protein double labeling by FISH and immunofluorescence assay

Before immunofluorescence assay, cells were added with prehybridization solution at 37 °C for 1 h. After removing the prehybridization solution, the hybridization solution containing the probe of *SNORA71* or *ROCK2* was added and hybridized overnight. After washing with SSC, cells were incubated with primary antibody anti‐G3BP1 (1 : 500; # ab181150; Abcam) and secondary antibody: primary antibody were incubated overnight at 4 °C, then washed with PBS for 3 × 5 min; Corresponding secondary antibody was dropped and incubated at room temperature for 50 min, then washed with PBS for 3 × 5 min. DAPI dye for dyeing nuclear for 8min away from light. Images were obtained using a fluorescence microscope.

### 2.22 Xenograft assay

MDA‐MB‐231 cells stably transduced with lentivirus vectors carrying sh‐*SNORA71*, sh‐NC, oe*‐SNORA71A,* or empty vectors were used for the establishment of the xenograft mouse model. Female 5‐ to 6‐week‐old Balb/c nude mice purchased from Wuhan Cloud Clone Animal Co., LTD (Wuhan, China), were used in this study. Mice were housed at the regular housing temperatures, under a constant 12‐h light/dark cycle with food and water available *ad libitum*. For overexpression, mice were randomly divided into two groups (*n* = 5): *SNORA71A* group and NC group, and for knockdown, mice were randomly divided into two groups (*n* = 6): sh‐*SNORA71* group and sh‐NC group. Cells were resuspended using PBS with 6 × 10^6^ cells in 100 μL PBS, and 100 μL of cell suspension was subcutaneously injected into mice. The experiments lasted for 3 weeks, and the tumor size of mice was detected every 3 days. Mice were euthanized by CO_2_ asphyxiation procedure. The tumor volume was evaluated as width^2^ × length × 0.5. The lung metastasis model was established by tail vein injection of 2 × 10^6^ sh‐NC and sh‐SNORA71 MDA‐MB‐231 cells suspended in 200 mL of PBS (*n* = 5). After 5 weeks of injection, lung tissues were isolated and photographed. The relative number of metastatic lung nodules of mice was counted. The animal experiments in this study and all procedures involving the handling and treatment of mice during this study were approved by the Laboratory Animal Welfare & Ethics Committee (IACUC) of Renmin Hospital of Wuhan University (Issue No. 20200702). All the experiments were performed according to the guidelines of the National Institutes of Health Guide for the Care and Use of Laboratory Animals.

### 2.23 Construction of receiver operating characteristic curve

The receiver operating characteristic (ROC) curve was plotted via the graphpad prism 8.0.1 to evaluate specificity and sensitivity of the snoRNA expression‐based diagnose signature, and *P* < 0.05 represented statistical significance.

### 2.24 Statistical analysis

Statistical analysis was carried out by graphpad prism 6 (GraphPad Software, La Jolla, CA, USA). Statistical analysis between two groups was performed by Student's *t*‐test (two‐tailed), and a one‐way ANOVA post‐Tukey test was applied for the comparison between more than two groups. All cell experiments were performed in three biological replicates. Significance was identified at *P* > 0.05, and data are shown as Mean ± SD in the figures.

## 3 Results

### 3.1 Expression profile of snoRNA in breast cancer EMT models

To establish an EMT model, breast cancer cells were stimulated with TGF‐β, which is a crucial molecule for the EMT process [15, 16]. TGF‐β stimulation resulted in the transition from epithelial to the mesenchymal‐like phenotype in both MCF‐7 and MDA‐MB‐231 cells (Fig. S1A). Moreover, TGF‐β increased the mRNA and protein levels of Vimentin, while decreased E‐cadherin in MCF‐7 and MDA‐MB‐231 cells (Fig. S1B–E). To obtain the expression pattern of snoRNAs, the EMT and control cells were sequenced and generated 13–21 million clean reads (Table S3). In average, 4.8 million reads per library were mapped to snoRNAs with 29.52% mapped rate and 17.3 unique mapped rate (Table S4). The length of snoRNAs was primarily ranged from 55 to 85 bp with a peak appeared at length of 71 bp (Fig. S2A). Moreover, 193 snoRNAs were identified, including 66 H/ACA box snoRNAs, 101 C/D box snoRNAs, and 26 unknown snoRNAs. The abundance of C/D box snoRNAs was the highest, accounting for 80.04% of the total counts (Fig. S2B).

### 3.2 *SNORA71A* is upregulated in metastatic breast cancer tissues and cells

**Fig. 1:**
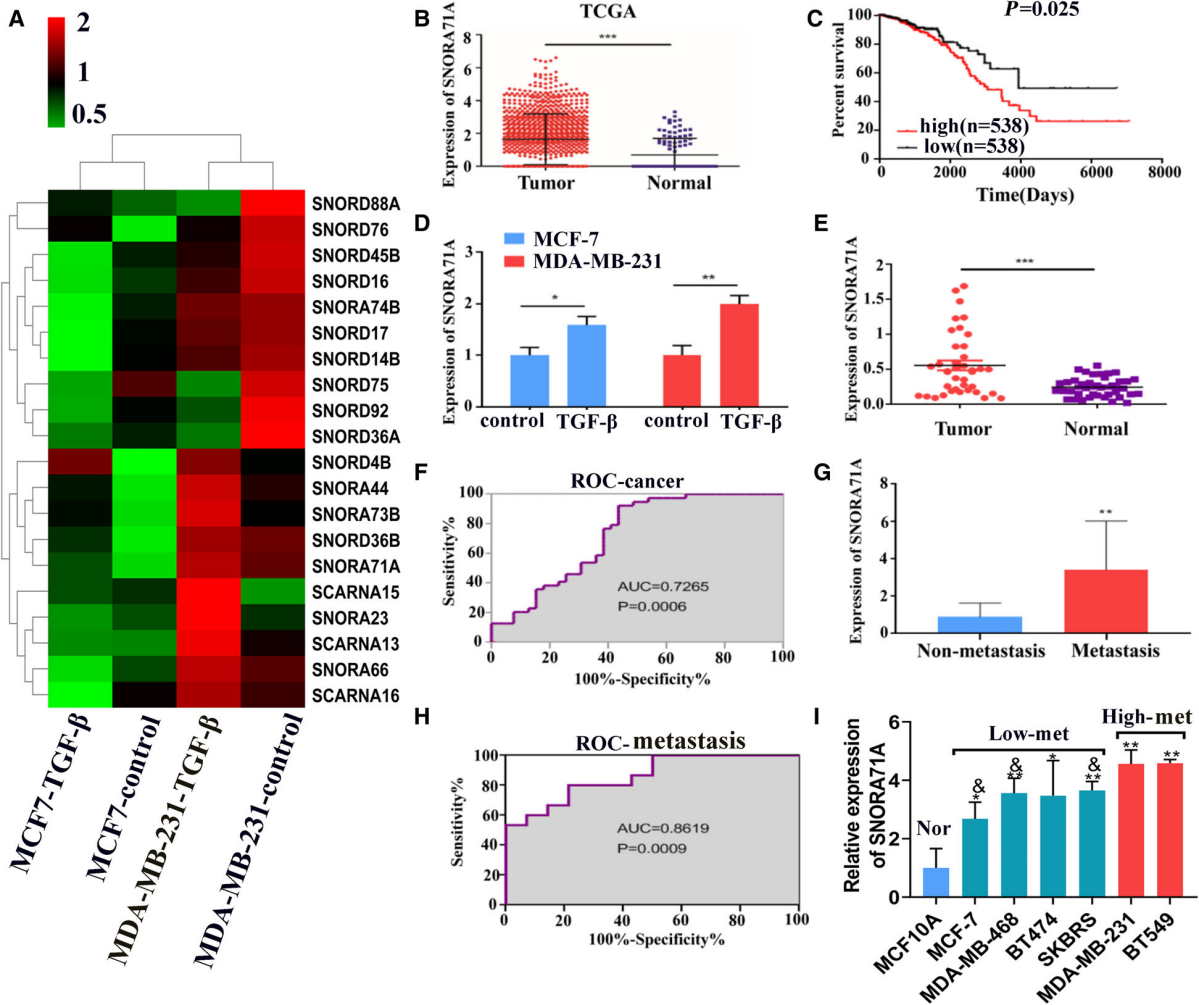
Expression of *SNORA71A* in breast cancer tissues and cells. (A) The heatmap shows all differentially expressed snoRNAs identified by snoRNA sequencing in TGF‐β‐induced MCF‐7 and MDA‐MB‐231 cells and their control cells (without any treatment). Red indicates high expression; green indicates low expression. (B) The expression of *SNORA71A* in breast cancer tissues retrieved from the TCGA database. Tumor (*n* = 1077), Normal (*n* = 105). Error was defined as SD. *t*‐test. (C) Analysis of the relationship between *SNORA71A* expression and survival of breast cancer patients was performed based on the TCGA database. (D) *SNORA71A* expression was verified by real‐time PCR in TGF‐β‐treated MCF‐7 and MDA‐MB‐231 cells and their control cells. *N* = 3, one‐way ANOVA. Error was defined as SD. (E) *SNORA71A* expression in breast cancer tissues and normal tissues adjacent to carcinoma was verified by real‐time PCR (39 biological replicates and 3 technical repeats); *t*‐test. Error was defined as SD. (F) ROC analysis of *SNORA71A* for diagnose of breast cancer (39 biological replicates). ROC curve analysis. (G) *SNORA71A* expression in metastatic (*n* = 15) and nonmetastatic tumor tissues (*n* = 14) from patients with breast cancer; *t*‐test. Error was defined as SD. (H) ROC analysis of *SNORA71A* for diagnose of metastasis. *N* = 14. ROC curve analysis. (I) *SNORA71A* expression was verified by real‐time PCR in human normal breast epithelial cells and breast cancer cells with different metastatic potential (*n* = 3); Error was defined as SD. *P < 0.05 compared with normal cells; ^&^ *P* < 0.05 compared with high metastatic cells. *N* = 3, one‐way ANOVA, *P < 0.05, **P < 0.01, ***P < 0.001.

A total of 20 differentially expressed snoRNAs were identified between the TGF‐β and control group, among which four snoRNAs were consistently upregulated (including *SNORA71A*) in both two cell lines, and two were commonly downregulated (Fig. 1A, Table S5). We verified the six snoRNAs that differentially expressed in both cell lines, and found the real‐time PCR data provide confidence in the sequencing data (Fig. S2C). To identify vital snoRNAs underlying the breast cancer metastasis, we further analyzed these snoRNAs using the TCGA database (http://bioinfo.life.hust.edu.cn/SNORic/download/). We found *SNORA71A* was also significantly upregulated in the breast cancer tumor tissues compared to the normal tissues (Fig. 1B). Surprisingly, high expression of *SNORA71A* correlated significantly with the poor prognosis of patients with breast cancer (*P* = 0.025, Fig. 1C). Real‐time PCR analysis confirmed the upregulated expression of *SNORA71A* in TGF‐β‐treated MCF‐7 and MDA‐MB‐231 cells compared with their corresponding parental cells (Fig. 1D).

Furthermore, real‐time PCR showed the expression of *SNORA71A* was significantly increased in the breast cancer tissues compared to the adjacent peritumoral tissues (Fig. 1E). Receiver operating characteristic (ROC) curve analysis showed *SNORA71A* might serve as a biomarker for breast cancer, with the area under curve (AUC) of 0.72 (*P* = 0.0006) (Fig. 1F). At the cutoff value, the sensitivity and specificity of breast cancer diagnosis were 76.92% and 61.54%, respectively (Fig. 1F). We further focused on the metastasis role of *SNORA71A* in breast cancer. *SNORA71A* was upregulated in the tumor tissues from the patients with metastasis compared to that without metastasis (Fig. 1G). ROC curve displayed that the tissue *SNORA71A* may serve as a biomarker for metastasis, with AUC of 0.86 (*P* = 0.0009) (Fig. 1H). At the cutoff value, the sensitivity and specificity of metastasis diagnosis were 60% and 92.86%, respectively.

When patients with breast cancer were divided into two groups with high (*n* = 20) or low *SNORA71A* expression (*n* = 19) according to the real‐time PCR analysis, a significant correlation between *SNORA71A* expression and the tumor stage, ER status and lymph node metastasis was observed (Table S1). While the *SNORA71A* expression was not significantly associated with clinical parameters of age, tumor grade, PR status, or HER‐2 expression status (Table S1).

Furthermore, the expression of *SNORA71A* was evaluated in normal breast epithelial cells and breast cancer cells with different metastasis potential. *SNORA71A* expression was increased in the breast cancer cells compared to that in the normal breast epithelial cells (Fig. 1I). Importantly, *SNORA71A* expression was remarkably increased in the highly metastatic breast cancer cell line MDA‐MB‐231 compared to that in the lowly‐metastatic breast cancer cell line MCF‐7.

### 3.3 *SNORA71A* promotes EMT process of breast cancer cells

**Fig. 2:**
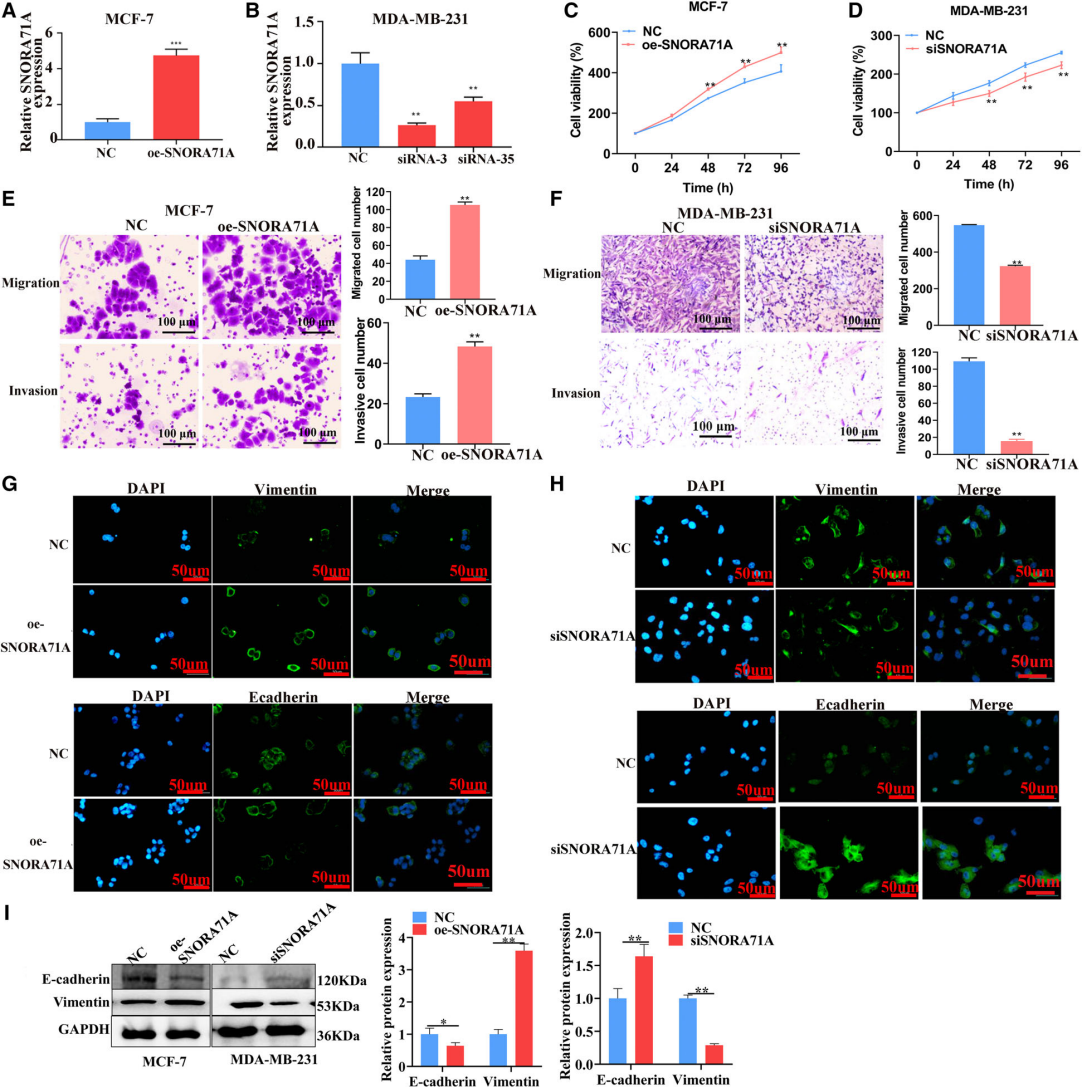
Effect of *SNORA71A* on breast cancer cells. (A) The overexpression of *SNORA71A* in MCF‐7 cells was verified by real‐time PCR after transfection for 24 h (*n* = 3). NC: empty vector; *t*‐test. Error was defined as SD. (B) The efficiency of *SNORA71A* silencing in MDA‐MB‐231 cells was verified by real‐time PCR after transfection for 24 h (*n* = 3). Error was defined as SD. NC: negative control siRNA. The *SNORA71A* siRNA‐3 was used for next study; *t*‐test. The Cts of expression upon SNORA71A overexpression and respective NC in MCF‐7 cells were 18 and 20, respectively; and SNORA71A silencing and respective NC were about 21 and 20. (C, D) The effect of *SNORA71A* on cell proliferation was detected by CCK‐8 assay (*n* = 6); *t*‐test. Error was defined as SD. (E, F) The effect of *SNORA71A* overexpression and silencing on migration and invasion was detected by transwell assay after transfection for 48 h in MCF‐7 and MDA‐MB‐231 cells, respectively. Scale bar = 100 μm; *t*‐test (*n* = 3). Error was defined as SD. (G, H) Immunofluorescence displays the expression of EMT marker in MCF‐7 cells (G) and MDA‐MB‐231 cells (H). Scale bar = 50 μm. (I) The effect of *SNORA71A* on the expression of the EMT marker was detected by western blotting after transfection for 48 h (*n* = 3). Error was defined as SD. NC for silencing was cells transfected with si‐control, NC for *SNORA71A* overexpression was cells transfected with empty vector. *T*‐test. *P < 0.05, **P < 0.01, ***P < 0.001.

To evaluate the role of *SNORA71A* in metastasis of breast cancer, we overexpressed and silenced *SNORA71A* in both MDA‐MB‐231 cells and MCF‐7 cells. Overexpression of *SNORA71A* significantly promoted cell viability, while silencing of *SNORA71A* inhibited cell viability in MCF‐7 and MDA‐MB‐231 cells (Fig. 2A–D and Fig. S3A,B). *SNORA71A* overexpression remarkably enhanced the migration and invasion ability of MCF‐7 and MDA‐MB‐231 cells (Fig. 2E and Fig. S3C), while the deficiency of *SNORA71A* led to the suppression of migration and invasion ability in both MDA‐MB‐231 and MCF‐7 cells (Fig. 2F and Fig. S3C). Interestingly, upregulation of *SNORA71A* decreased immunofluorescence signal of epithelial marker E‐cadherin and increased that of mesenchymal marker Vimentin in MCF‐7 cells (Fig. 2G). Conversely, the downregulation of *SNORA71A* increased the E‐cadherin expression and decreased the Vimentin expression in MDA‐MB‐231 cells (Fig. 2H). Similarly, western blotting revealed *SNORA71A* suppressed the E‐cadherin expression and promoted the Vimentin expression (Fig. 2I and Fig. S3D). Moreover, *SNORA71A* suppressed cell apoptosis of MCF‐7 and MDA‐MB‐231 cells (Fig. S4). The Edu assay verified *SNORA71A* promoted the proliferation of breast cancer cells (Fig. S5A). However, upregulation or downregulation of *SNORA71A* had no significant effect on the cell cycle (Fig. S5B).

Since there is an effect of *SNORA71A* on proliferation, to prevent confounding effects, the migration and invasion experiments were also conducted in the presence of aphidicolin (the final concentration was 1 mg·L^−1^). The results showed in the presence of aphidicolin, *SNORA71A* also promoted the migration and invasion ability of breast cancer cells (Fig. S6).

Additionally, another siRNA was also used to verify the promote effect of *SNORA71A* on migration, invasion, and EMT (Fig. S7). We also tested whether *SNORA71A* expression is necessary for TGF‐β‐mediated EMT, by knocking‐down *SNORA71A* and treating cells with TGF‐β. The results showed deficiency of *SNORA71A* significantly abrogated the promote effect of TGF‐β on EMT (Fig. S8).

### 3.4 *SNORA71A* upregulates *ROCK2* in the TGF‐β signaling pathway

**Fig. 3:**
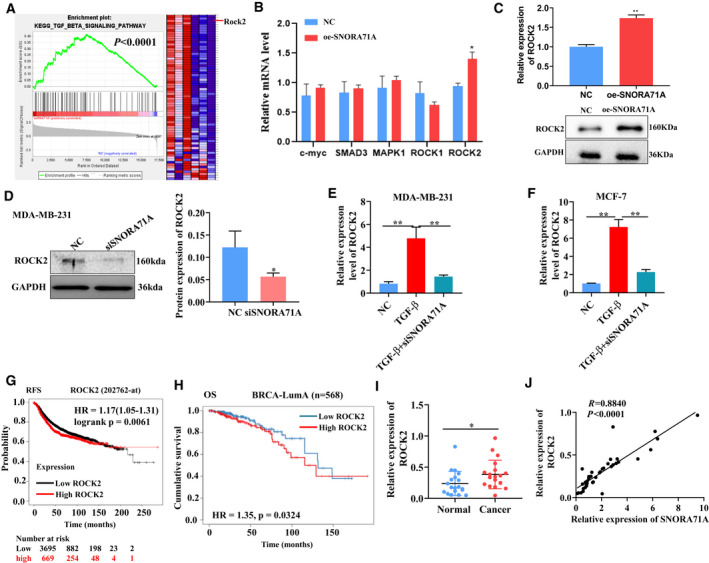
RNA‐seq reveals the genes and pathways related to *SNORA71A*. (A) Gene Set enrichment analysis (GSEA) for the genes identified by RNA‐seq in DA‐MB‐231 cells transfected with *SNORA71A*‐overexpressing or empty vector for 24 h. Three biological replicates. The GSEA was performed using all genes (including genes that did not differ significantly), and the red color in the GSEA only represents relatively high expression level, but not represents upregulated. (B) The mRNA expression of genes enriched in TGF‐β signaling was evaluated by real‐time PCR in MDA‐MB‐231 cells. Error was defined as SD. NC: empty vector. oe‐SNORA71A: *SNORA71A*‐overexpressing plasmid. Cells were transfected with for 24 h. *N* = 3; *t*‐test. (C) The expression of *ROCK2* was verified by western blot in MDA‐MB‐231 cells (*n* = 3). Error was defined as SD. NC: empty vector. oe‐SNORA71A: *SNORA71A*‐overexpressing plasmid. Cells were transfected with for 48 h; *t*‐test. (D) The expression of ROCK2 protein was measured by western blot in MDA‐MB‐231 cells. NC: cells were transfected by si‐control for 48 h. Error was defined as SD. *N* = 3, *t*‐test. *P < 0.05. (E, F) The expression of *ROCK2* was measured by real‐time PCR in MDA‐MB‐231 or MCF‐7 cells. NC: cells without any treatment. TGF‐β: cells were only treated with TGF‐β for 48 h, and without si‐control transfected. TGF‐β+siSNORA71A: cells were transfected with siSNORA71A and treated with TGF‐β for 48 h. Error was defined as SD. *N* = 3, one‐way ANOVA. **P < 0.01. (G) Relationship of *ROCK2* expression and recurrence‐free survival (RFS) of breast cancer patients was analyzed by Kaplan–Meier Plotter database (Affy ID: 202762_at). Kaplan–Meier statistical analysis. (H) Relationship of *ROCK2* expression and over survival (OS) of BRCA‐LumA patients was analyzed by Timer 2.0 database. Kaplan–Meier statistical analysis. (I) The expression of *ROCK2* in breast cancer tissues and normal tissues adjacent to carcinoma was verified by real‐time PCR (*n* = 19). Error was defined as SD; *t*‐test. (J) The correlation of *SNORA71A* and *ROCK2* expression as analyzed by real‐time PCR (*n* = 19). Pearson correlation. *P < 0.05.

To investigate the genes and pathways involved in *SNORA71A‐*mediated EMT, we performed mRNA‐seq in *SNORA71A*‐overexpressing cells and control cells. GSEA analysis using the complete set of identified genes revealed that TGF‐β signaling was the most enriched pathway (Fig. 3A). Among the genes enriched in the TGF‐β signaling, *ROCK2* was the most significantly enriched gene. Additionally, genes, including *ROCK1, Smad3, MYC* and *MAPK1* that have been generally known to function in neoplastic processes, were also significantly enriched in the TGF‐β signaling. To investigate whether *SNORA71A* is involved in the TGF‐β‐mediated EMT by regulating these genes, we verified their expression by real‐time PCR in *SNORA71A*‐overexpressing cells. Results revealed that high expression of *SNORA71A* significantly increased the mRNA and protein levels of *ROCK2* (Fig. 3B,C). Similarly, silencing of *SNORA71A* significantly decreased the expression of *ROCK2* (Fig. 3D). To clearly establish the importance of *SNORA71A* in induction of ROCK2 during TGF‐β stimulation, TGF‐β stimulation in combination with and without siSNORA71A was performed, with the measurement of *ROCK2*. Results showed *SNORA71A* significantly induced *ROCK2* during TGF‐β stimulation in MCF‐7 and MDA‐MB‐231 cells (Fig. 3E,F). Furthermore, using the Kaplan–Meier Plotter and Timer 2.0 database, we found that patients with high expression of *ROCK2* had lower recurrence‐free survival (RFS) and overall survival (OS) time compared to those with low expression of *ROCK2* (Fig. 3G,H). Real‐time PCR showed *ROCK2* was increased in breast cancer tissues compared to normal tissues adjacent to carcinoma, and showed a significantly positive correlation with *SNORA71A* expression (Fig. 3I,J).

### 3.5 *SNORA71A* controls breast cancer EMT by *ROCK2*

**Fig. 4:**
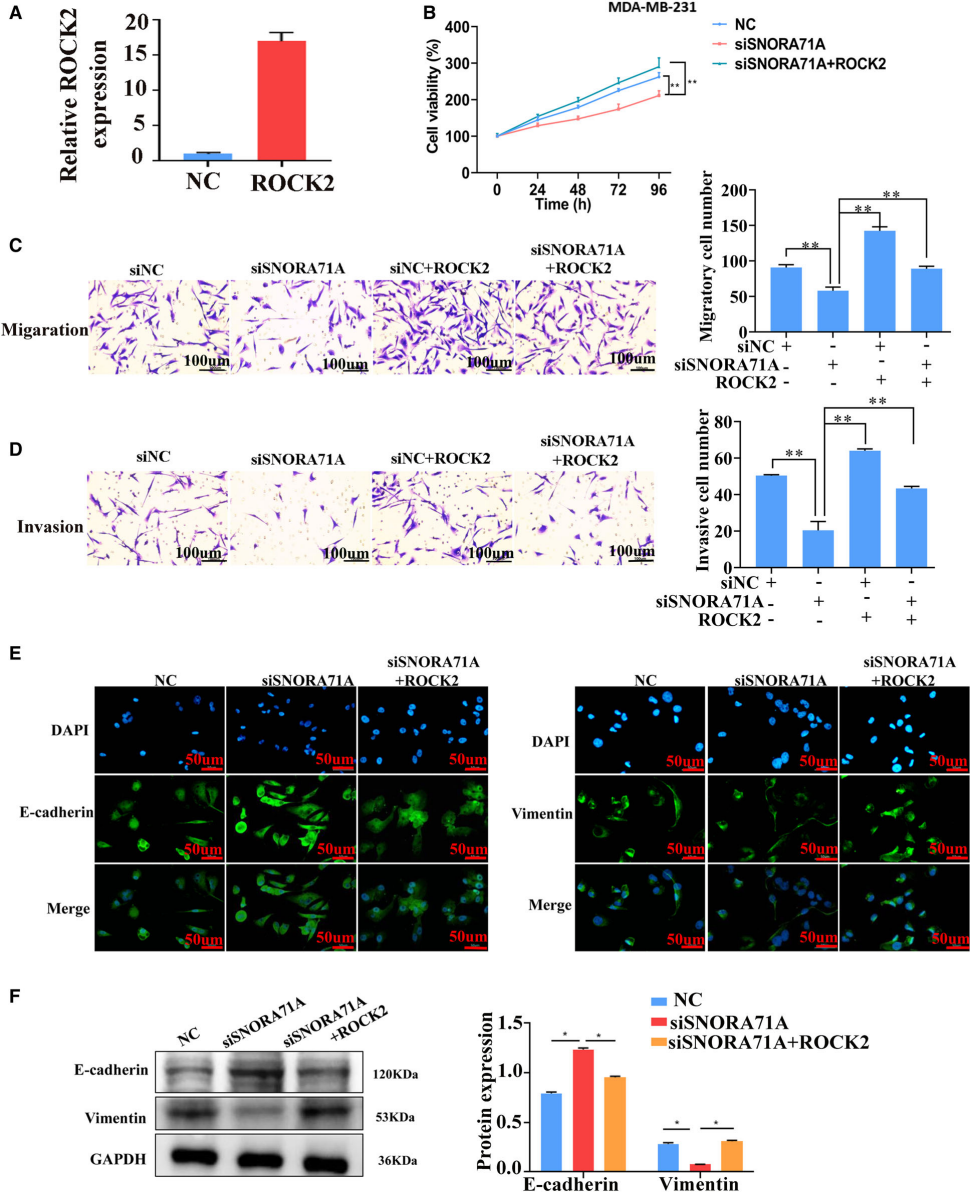
*SNORA71A* controls the breast cancer cells by *ROCK2*. (A) The efficiency of *ROCK2* overexpression in MDA‐MB‐231 cells was verified by real‐time PCR (*n* = 3). NC: empty vector, *t*‐test. (B) Cell proliferation was measured by CCK‐8 assay (*n* = 6). NC: cells were transfected by si‐control, one‐way ANOVA. (C, D) Migration and invasion of MDA‐MB‐231 cells was measured by transwell assay (*n* = 3). siNC: cells were transfected by si‐control, one‐way ANOVA. Scale bar = 100 μm. (E) Expression of EMT markers was measured by immunofluorescence in MDA‐MB‐231 cells (*n* = 3). Scale bar = 50 μm. (F) EMT markers were measured by western blotting in MDA‐MB‐231 cells. NC: cells were transfected by si‐control, one‐way ANOVA (*n* = 3), *P < 0.05, ***P < 0.001.

To investigate whether *ROCK2* mediated the function of *SNORA71A*, we firstly performed the invasion and migration experiments with *ROCK2* overexpression only. Results showed *ROCK2* was successfully overexpressed using the pcDNA 3.1 vector in MDA‐MB‐231 cells (Fig. 4A), and the *ROCK2* itself could promote the migration and invasion of breast cancer cells (Fig. S9). Although *SNORA71A* silencing significantly inhibited the proliferation of MDA‐MB‐231 cells, high levels of *ROCK2* remarkably restored the cell proliferating potential (Fig. 4B, Fig. S10). At *SNORA71A* deficiency, the migration and invasion of MDA‐MB‐231 cells were remarkably inhibited, while overexpression of *ROCK2* significantly abrogated this effect (Fig. 4C,D). Importantly, overexpression of *ROCK2* significantly reversed the increase of E‐cadherin and the decrease of Vimentin induced by *SNORA71A* deficiency (Fig. 4E,F). Furthermore, we overexpressed *SNORA71A* and knockdown *ROCK2* to further verify the *SNORA71A* controls breast cancer by *ROCK2*. We observed that *ROCK2* knockdown significantly blocked *SNORA71A* to enhance the proliferation, migration, invasion and EMT of MDA‐MB‐231 cells (Fig. S11). These data indicate that *SNORA71A* might control EMT progress of breast cancer cells, partly by regulating *ROCK2*.

### 3.6 *SNORA71A* upregulates *ROCK2* by mRNA stability regulatory protein G3BP1

**Fig. 5:**
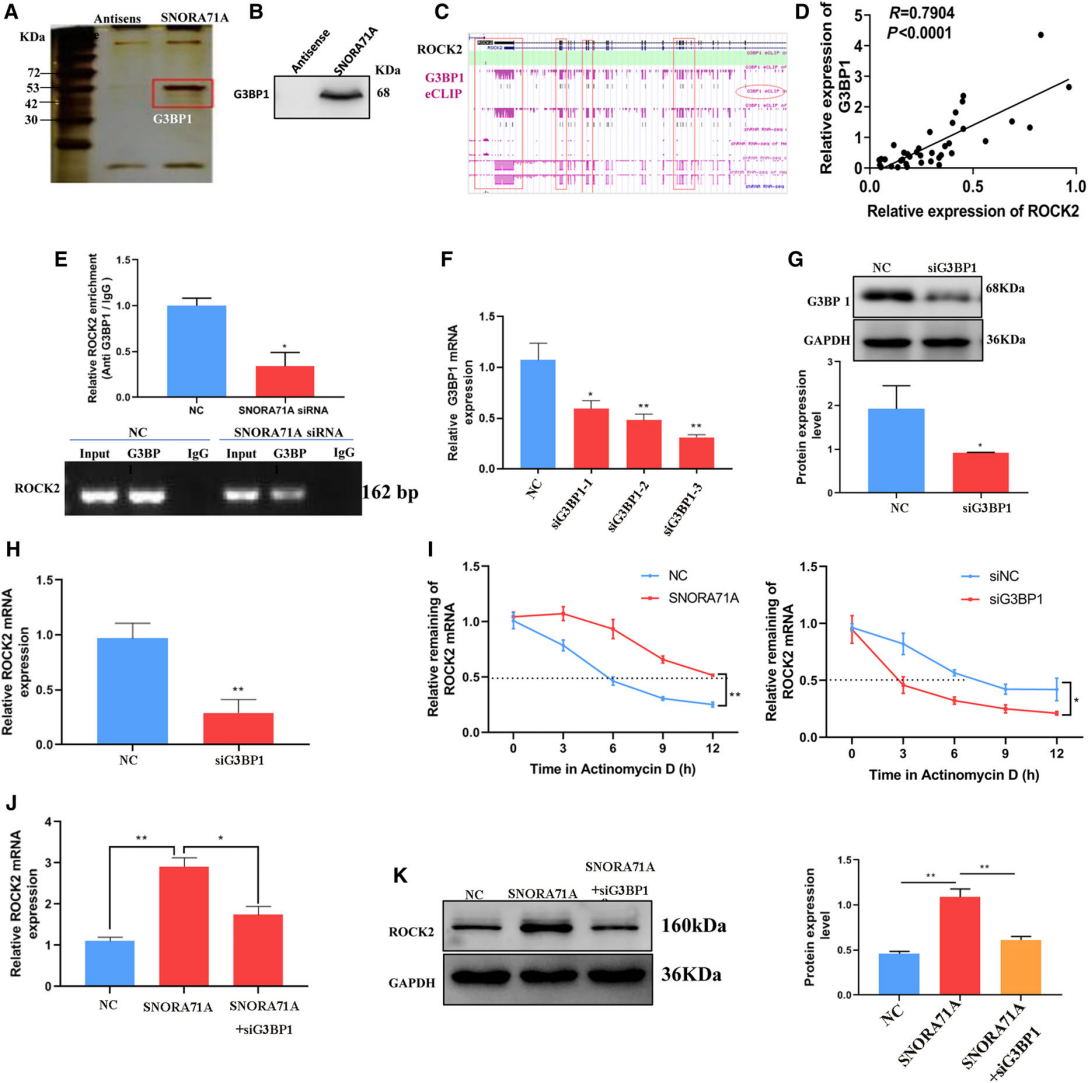
*SNORA71A* upregulates *ROCK2* by binding to G3BP1. (A) SDS/PAGE protein gel silver staining of *SNORA71A* pull‐down products (one biological replicate). The red‐framed bands represent specific bands that were subjected to mass spectrum. G3BP1 was found by the mass spectrum analysis. (B) Western blotting was used to verify the binding of *SNORA71A* to G3BP1 (one biological replicate). (C) Screenshot shows significant binding of G3BP1 protein to mRNA of *ROCK2*, based on ENCODE database. (D) GEPIA database shows that G3BP1 expression is positively correlated with *ROCK2* expression in breast cancer. (E) RIP assay with anti‐G3BP1 antibody was performed in the MDA‐MB‐231 cells. NC: cells were transfected with si‐control for 48 h (*n* = 3). Error was defined as SD. The column diagram shows the enrichment of *ROCK2* by real‐time PCR of G3BP1 RIP product; *t*‐test. (F) Real‐time PCR shows the efficiency of interference of G3BP1 after transfection for 24 h (*n* = 3). Error was defined as SD. The fragment, which showed the best interference, was used for further studies. NC: cells were transfected with si‐control, one‐way ANOVA. (G) Western blot analysis shows the efficiency of interference with the G3BP1 siRNA‐3 after transfection for 48 h (*n* = 3). Error was defined as SD. NC: cells were transfected with si‐control, *t*‐test. (H) Real‐time PCR shows the expression of *ROCK2* in MDA‐MB‐231 cells. Error was defined as SD. NC: cells were transfected with si‐control for 48 h, *t*‐test (*n* = 3). (I) *ROCK2* mRNA stability was determined by Real‐time PCR after Actinomycin D treatment (*n* = 3). Error was defined as SD, *t*‐test. NC: cells were transfected with empty vector. siNC: cells were transfected with si‐control. (J) Real‐time PCR shows the expression of *ROCK2* in MDA‐MB‐231 cells transfected with NC, *SNORA71A*, or *SNORA71A*+G3BP1 siRNA. NC: cells were transfected with empty vector, one‐way ANOVA (*n* = 3), Error was defined as SD. *P < 0.05. (K) Western blotting shows the expression of *ROCK2* after transfection for 48 h (*n* = 3). Error was defined as SD. NC: cells were transfected with empty vector, one‐way ANOVA, *P < 0.05, **P < 0.01.

**Fig. 6:**
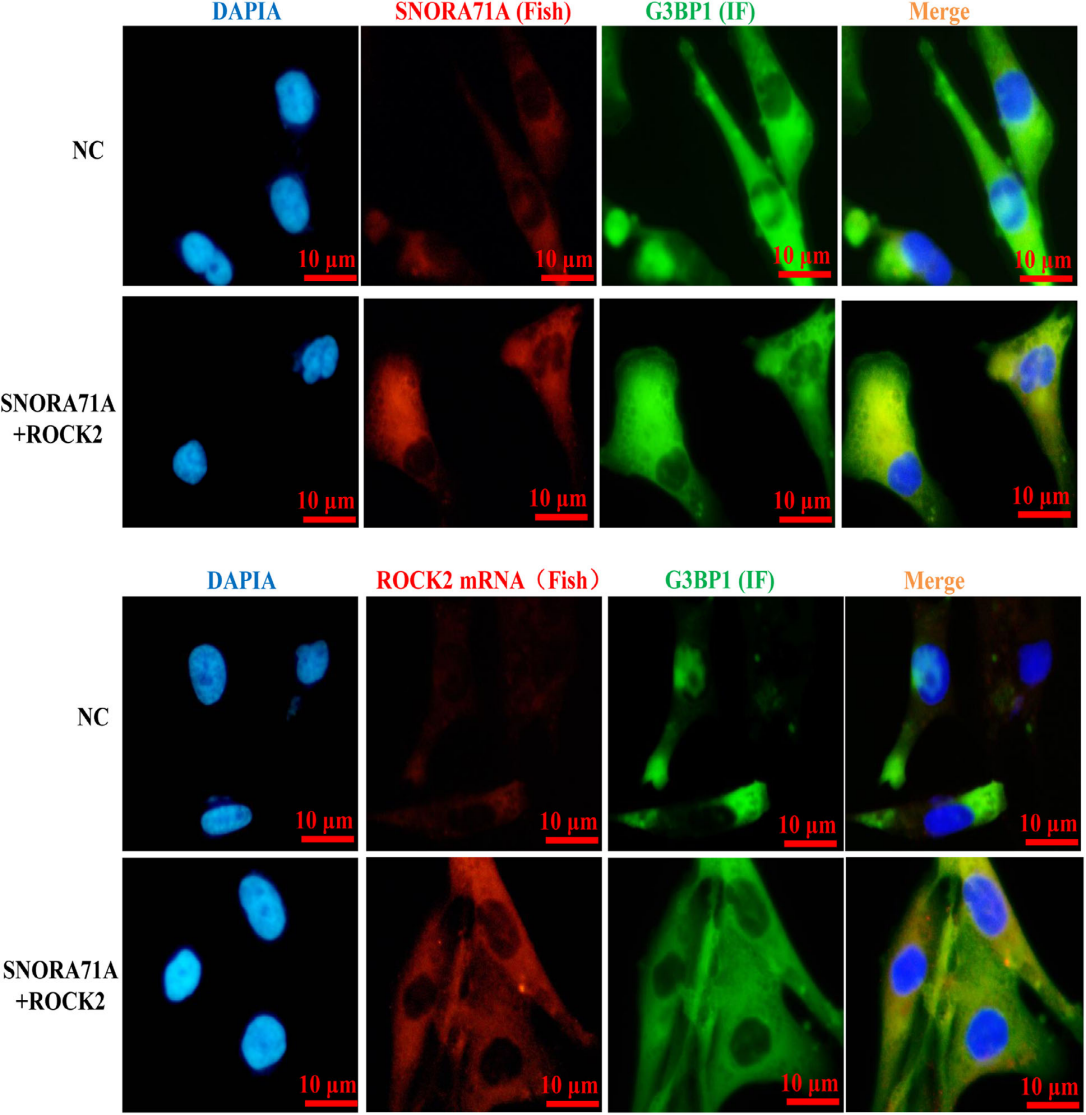
Location of *SNORA71A*, G3BBP1, and *ROCK2* in breast cancer cells. Double labeling of RNA–protein was performed by FISH and immunofluorescence (IF). The location of *SNORA71A* and *ROCK2* mRNA was detected by FISH, and the location of G3BP1 was detected by IF in MDA‐MB‐231 cells. Blue represents the nucleus, green represents G3BP1, and red represents *SNORA71A* or *ROCK2* mRNA, yellow represents co‐location. NC: cells were transfected with empty vector, *n* = 3, Scale bar = 10 μm.

To determine the mechanism by which *SNORA71A* mediates the expression of *ROCK2*, we performed RNA pull‐down and mass spectrum assays to uncover the binding proteins of *SNORA71A*. Compared to the control antisense probe, *SNORA71A* specifically binds to histones, translation initiation factors, and several RNA‐binding proteins, such as G3BP1 (Fig. 5A, Table S6). The binding of *SNORA71A* to G3BP1 was also verified by western blotting (Fig. 5B). Based on the eCLIP‐seq database, we found that G3BP1, which has been shown to regulate mRNA stability [17, 18], was significantly bound to the mRNA of *ROCK2* (Fig. 5C). Moreover, the GEPIA database showed that the expression of G3BP1 had a significant positive correlation with the expression of *ROCK2* in breast cancer (Fig. 5D). Therefore, we further investigated whether *SNORA71A* mediates the binding of G3BP1 and *ROCK2*, thus regulating the mRNA stability of *ROCK2*. RIP‐PCR assay displayed that G3BP1 could bind to the mRNA of *ROCK2*, while *SNORA71A* silencing significantly inhibited their binding activity (Fig. 5E). Interestingly, the deficiency of G3BP1 strongly decreased the mRNA level of *ROCK2* in MDA‐MB‐231 cells (Fig. 5F–H). We next used Actinomycin D to examine the effect of *SNORA71A* on the stability of *ROCK2* mRNA. As expected, overexpression of *SNORA71A* remarkably increased the mRNA stability of *ROCK2*, while silencing of G3BP1 showed the opposite effect (Fig. 5I). Rescue assay showed that overexpression of *SNORA71A* increased the mRNA and protein levels of *ROCK2*, whereas G3BP1 silencing remarkably abrogated this regulatory function of *SNORA71A* (Fig. 5J,K). Fish and immunofluorescence assays showed *SNORA71A* mainly located in the cytoplasm, and co‐located with G3BP1 protein (Fig. 6). Moreover, *ROCK2* mRNA and G3BP1 protein were also co‐located in cytoplasm, and both overexpression of *SNORA71A* and *ROCK2* increased the signal of *SNORA71A* and *ROCK2*, but has no effect on their location. Taken together, *SNORA71A* might recruit mRNA stability‐regulated protein G3BP1 to bind to the mRNA of *ROCK2*, thus enhancing the mRNA stability of *ROCK2*.

### 3.7 *SNORA71A* promotes tumor growth *in vivo*

**Fig. 7:**
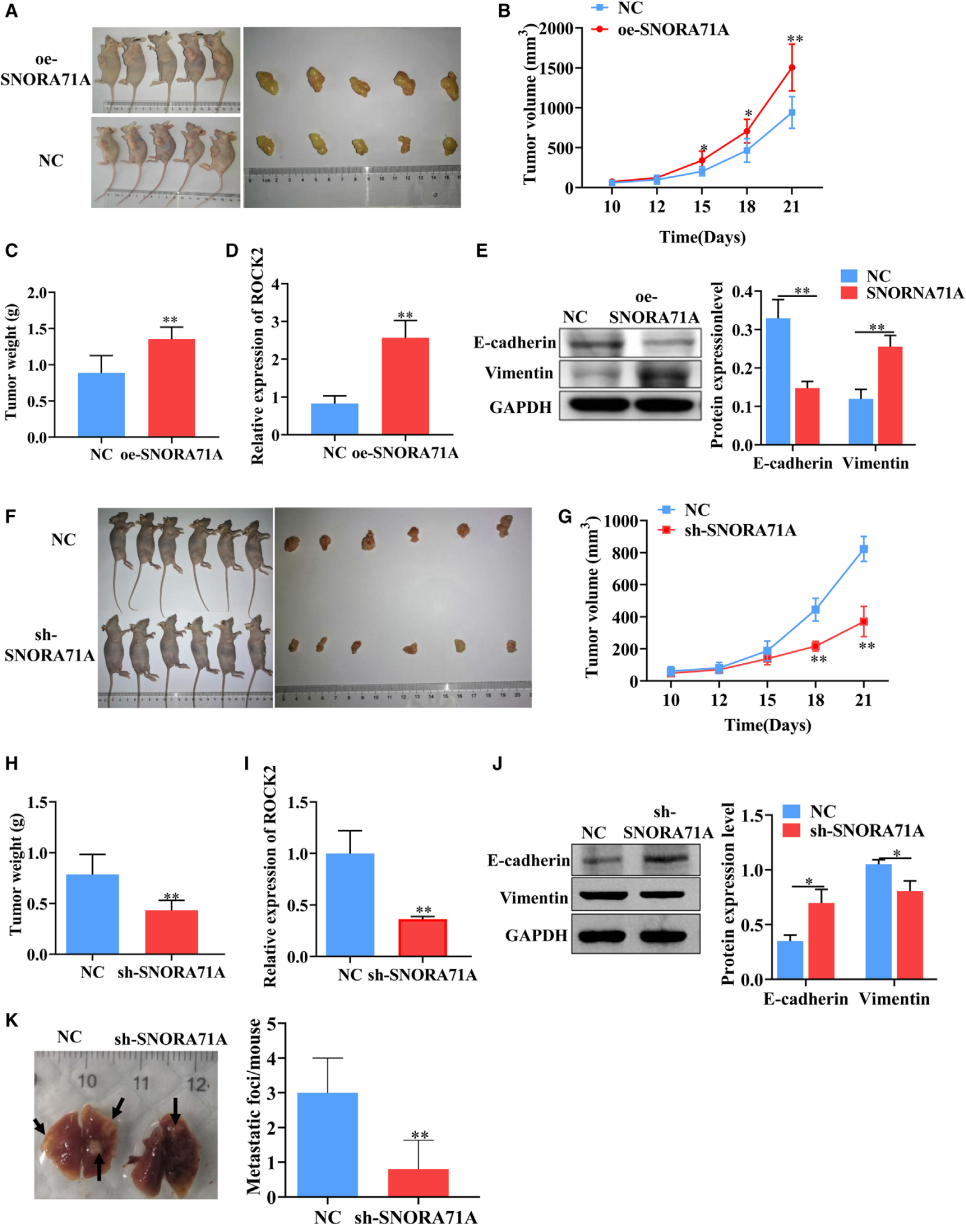
*SNORA71A* promotes tumor growth *in vivo*. (A) Images show the treated mice and the tumor tissues. Mice were subcutaneously injected with MDA‐MB‐231 cells (*n* = 5). NC: empty vector; oe‐*SNORA71A*: *SNORA71A*‐overexpressing. (B) The tumor volume of mice with *SNORA71A* overexpression or empty vector. (C) The tumor weight of mice with *SNORA71A* overexpression or empty vector, *t*‐test. (D) The mRNA expression of *ROCK2* in tumor tissues was detected by real‐time PCR (*n* = 3). *T*‐test. (E) The expression of EMT markers was measured by western blotting (*n* = 3). *T*‐test. (F) Images of mice subcutaneously injected with MDA‐MB‐231 cells (*n* = 6). NC: sh‐NC; sh‐*SNORA71A*: cells stably transfected with *SNORA71A* shRNA. (G) The tumor volume of mice with sh‐NC or sh*‐SNORA71A*, *t*‐test. (H) The tumor weight of mice with sh‐NC or sh*‐SNORA71A, t*‐test. (I) The mRNA expression of *ROCK2* in tumor tissues was detected by real‐time PCR (*n* = 3), *t*‐test. (J) The expression of EMT markers was measured by western blotting (*n* = 3), *t*‐test. (K) Representative images and the number of lung metastatic nodules of mice. Five biological replicates. Statistical significance was evaluated using *t*‐test, *P < 0.05, **P < 0.01.

To verify the carcinogenesis of *SNORA71A in vivo*, we injected mice with *SNORA71A*‐overexpressing MDA‐MB‐231 cells (Fig. 7A). The tumor weight and tumor volume were significantly increased in the *SNORA71A*‐overexpressing mice compared to the control mice (Fig. 7B,C). Notably, the mRNA levels of *ROCK2* were significantly increased in the tumor tissues of mice injected with *SNORA71A*‐overexpressing MDA‐MB‐231 cells compared to those in the control mice (Fig. 7D). High expression of *SNORA71A* significantly suppressed the E‐cadherin and increased Vimentin in the tumor tissues of mice (Fig. 7E). However, knockdown of *SNORA71A* has an opposite effect on the tumor size and the expression of *ROCK2* and EMT markers (Fig. 7F–J). We next investigated whether knockdown of *SNORA71A* affect migration and invasion *in vivo*. The lung metastasis model was established by tail vein injection of sh‐NC and sh‐*SNORA71A* MDA‐MB‐231cells, and the pictures of lung tissues were taken after 5 weeks. Knockdown of *SNORA71A* notably decreased the metastasis nodules of breast cancer cells in *in vivo* (Fig. 7K).

## 4 Discussion

In cancer, malignant cells can escape the primary tumor through EMT, invade surrounding tissues, and colonize distant sites through the blood or lymph pathways, resulting in metastasis [19]. The expression profiles and roles of snoRNAs in the EMT program remain unclear. In the present study, we first revealed the snoRNA expression profiles in EMT‐activated breast cancer cells and identified a snoRNA that functions as a positive regulator of EMT by upregulating *ROCK2* expression in the TGF‐β signaling pathway.

Emerging evidence implicates that snoRNAs play a pivotal role in multiple physiological processes and diseases, including cancer. For example, *SNORD50A* and *SNORD50B* inhibit tumorigenesis by directly binding and inhibiting K‐Ras in human cancer [20]. *SNORD89* enhances tumorigenesis via mediating the Notch1/c‐Myc pathway in patients with ovarian cancer [21]. *SNORD78* functions as an oncogene by increasing the proliferation of non‐small cell lung cancer cells via activation of G0/G1 cell cycle arrest [22]. Interestingly, the expression levels of C/D box snoRNAs are remarkably related to the frequency of leukemia stem cells in the patients with primary acute myelogenous leukemia [11]. In our study, we observed that *SNORA71A* was upregulated in the metastasis tissues and MDA‐MB‐231cells, which has high metastatic potential, and promoted the proliferation, migration, invasion and EMT in breast cancer cells. Similarly, *SNORA71A* drives proliferation, invasion, and migration of lung tumors by regulating the phosphorylation of MEK and ERK1/2 [13]. These data showed that *SNORA71A* promoted EMT development and might act as a therapeutic target for metastasis in breast cancer.

snoRNAs have been implicated in multiple regulatory mechanisms, including rRNA processing, RNA splicing, translation regulation, and oxidative stress response. For instance, snoRNA HBII‐52 targets a silent element of the exon via complementary base‐pairing and mediates the selective splicing of 5‐ht2cr [23, 24]. snoRNAs preferentially bind and interact with the DNA‐binding domain of PARP‐1 and stimulate catalytic activity of PARP‐1 to promote cell proliferation [25]. The most classic mechanism of snoRNA activity is control of rRNA processing and biogenesis by guiding the modifications on rRNA positions [26]. In addition to guiding rRNA modification, snoRNAs also promote 2′‐*O*‐methylation modification on mRNA, which increases the expression of mRNA while inhibits the translation [10]. In this study, we found that *SNORA71A* overexpression elevates *ROCK2*, which can rescue the EMT, mediated by *SNORA71A*. *ROCK2* is a member of the TGF‐β signaling and has been widely reported to directly regulate the EMT program in cancer [27, 28, 29, 30]. Therefore, we speculate that *SNORA71A* is involved in the TGF‐β‐induced EMT by *ROCK2*.

**Fig. 8:**
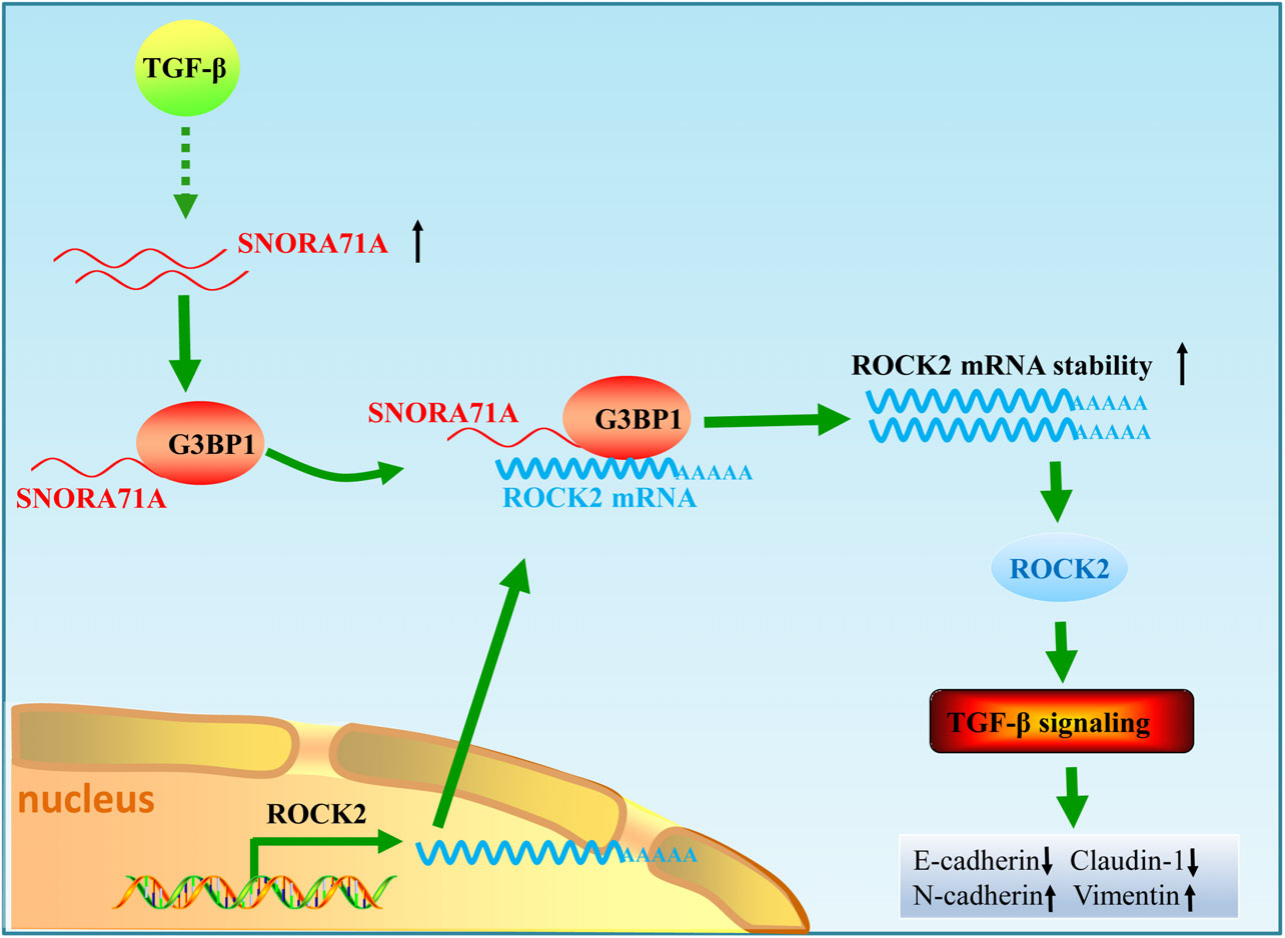
Molecular mechanisms of *SNORA71A*‐regulating EMT in breast cancer. *SNORA71A* recruits G3BP1 to the mRNA of *ROCK2*, thus increasing the mRNA stability of *ROCK2*, promoting thereby the EMT development of breast cancer by TGF‐β signaling.

The mechanism, by which *SNORA71A* regulates *ROCK2*, may implicate the aforementioned snoRNA mechanisms. For instance, *SNORA71A* may regulate the processes of *ROCK2*‐related ribosomes or directly modulate *ROCK2* mRNA modification, processing, or stability [10, 31]. We exclude the mechanism of *SNORA71A*‐mediated *ROCK2* regulation through the microRNA‐like functions [32], due to its activating effect on *ROCK2*. We found *SNORA71A* can bind to RNA‐binding protein G3BP1, and promote the binding of G3BP1 to *ROCK2* mRNA. Moreover, silencing of G3BP1 abolished the upregulatory effect of *SNORA71A* on *ROCK2*. Previous studies have shown that G3BP1 mediates mRNA stability [17, 18]. Moreover, G3BP1 has been implicated to be involved in the TGF‐β/Smad and p53 signaling pathways, and contribute to tumor progression and metastasis [33, 34]. These evidences support one of the S*NORA71A* mechanisms that it might recruit G3BP1 to the mRNA of *ROCK2*, thus increasing the mRNA stability of *ROCK2*, promoting thereby the EMT development of breast cancer by TGF‐β signaling (Fig. 8). Interestingly, we also found *SNORA71A* could bind to VIM by MS. Previous studies have reported that RNA, such as circular RNA [35], can directly bind to VIM. At present, there is no literature report that snoRNA can bind to VIM. We speculate that *SNORA71A* may promote the EMT also via directly regulating VIM protein. Nevertheless, the detailed mechanisms of *SNORA71A* activity in metastasis development need to be further investigated.

In the present study, TGF‐β dramatically activated the expression of *SNORA71A*. There are many transcription factors downstream of TGF‐β, including Smad3, Sp1, and Myc. It has been reported that TGF‐β can activate the expression of noncoding RNA, for instance, TGF‐β can activate lncRNA LINC00115, which is a critical regulator for glioma stem‐like cell tumorigenicity [36]. We hypothesized that TGF‐β may promote the transcription of *SNORA71A* through the downstream transcription factors. However, future research will further clarify how TGF‐β‐induces *SNORA71A* expression.

Molecular biomarkers provide an effective method for the early detection of breast cancer and contribute to personalized treatment for patients. Currently, multiple biomarkers have been developed and used as routine prognostic markers to identify cancer types and guide treatment in breast cancer, for instance, TP53 mutation, immune biomarkers (programmed death‐ligand 1 (PDL1)), and breast cancer susceptibility gene 1 or 2 (BRCA1/2) and PI3K/AKT/mTOR [37]. However, the incidence of recurrence, distant organ metastasis and breast cancer‐related death after treatment remains high. Therefore, it is urgent to find new biomarkers and molecular therapeutic targets. Currently, noncoding RNA has been reported as a diagnostic marker for breast cancer [38, 39]. The role of snoRNAs in breast cancer remains unknown. We showed *SNORA71A* could distinguish between normal samples and tumor samples, as well as patients with nonmetastatic breast cancer and patients with metastasis, indicating that *SNORA71A* might act as a novel biomarker for breast cancer.

## 5 Conclusion

In summary, we demonstrated that *SNORA71A* was upregulated in the breast cancer tissues and were associated with poor prognosis of breast cancer patients. *SNORA71A* promotes tumor growth and metastasis in breast cancer. Moreover, *SNORA71A* increases the mRNA stability of *ROCK2* via binding to G3BP1. Chemically modified anti‐*SNORA71A* agents, suppressing the metastasis of breast cancer, may have therapeutic potential and represent a novel strategy for treatment against metastasizing cancers.

## Conflict of interest

The authors declare no conflict of interest.

## Author contributions

QW and CC conceived and designed the experiments. TH and CL performed the main experiments and analyzed the data. YX, LW, and JS participated in cell survival and migration assay. TH, QW, CC, and CL wrote the manuscript. All authors read and approved the final manuscript.

## Supporting information


**Fig. S1.** Establishment of EMT model in breast cancer cells.
**Fig. S2.** Expression profiles of snoRNA in TGF‐β‐induced breast cancer cells.
**Fig. S3.** *SNORA71A* promotes cell proliferation, migration, invasion and EMT in breast cancer cells.
**Fig. S4.** *SNORA71A* inhibits cell apoptosis in breast cancer cells.
**Fig. S5.** Effect of *SNORA71A* on cell proliferation and cell cycle.
**Fig. S6.** The migration and invasion experiments were also conducted in the presence of aphidicolin (1mg/L).
**Fig. S7.** Effect of *SNORA71A* siRNA‐2 on migration, invasion and EMT.
**Fig. S8.** Deficiency of *SNORA71A* abrogated the promote effect of TGF‐β on EMT.
**Fig. S9.** *ROCK2* promotes the migration and invasion of breast cancer cells.
**Fig. S10.** *ROCK2* overexpression significantly blocked the silencing of *SNORA71A* to enhance the proliferation of breast cancer cells.
**Fig. S11.** Silencing of *ROCK2* blocks the increased effect of *SNORA71A* in cell proliferation, migration, invasion and EMT of MDA‐MB‐231 cells.
**Table S1.** Clinical features of breast cancer patients, and the correlationship between *SNORA71A* expression and different clinical features.
**Table S2.** Primer and siRNA sequences used in this study.
**Table S3.** Statistic analysis ofreads and base from snoRNA sequencing data.
**Table S4.** Mapping statistics of snoRNA from small RNA sequencing data.
**Table S5.** The commonly upregulated and downregulated snoRNA in both two breast cancer cells.
**Table S6.** Mass spectrometry of *SNORA71A* pull down product. The“Entry name” shows theproteins may bind to *SNORA71A*.

## Data Availability

The datasets used and/or analyzed during the current study are available from the corresponding author on reasonable request.
