# Supplementary material for: Small nucleolar RNA SNORA71A promotes epithelial‐mesenchymal transition by maintaining ROCK2 mRNA stability in breast cancer
Source: Mol Oncol. 2022 Mar 30;16(9):1947–65. doi: 10.1002/1878-0261.13186 (PMC9067147; doi:10.1002/1878-0261.13186)
Supplement: Supplementary file 1 — Fig. S1. Establishment of EMT model in breast cancer cells. Fig. S2. Expression profiles of snoRNA in TGF‐β‐induced breast cancer cells. Fig. S3. SNORA71A promotes cell proliferation, migration, invasion and EMT in breast cancer cells. Fig. S4. SNORA71A inhibits cell apoptosis in breast cancer cells. Fig. S5. Effect of SNORA71A on cell proliferation and cell cycle. Fig. S6. The migration and invasion experiments were also conducted in the presence of aphidicolin (1mg/L). Fig. S7. Effect of SNORA71A siRNA‐2 on migration, invasion and EMT. Fig. S8. Deficiency of SNORA71A abrogated the promote effect of TGF‐β on EMT. Fig. S9. ROCK2 promotes the migration and invasion of breast cancer cells. Fig. S10. ROCK2 overexpression significantly blocked the silencing of SNORA71A to enhance the proliferation of breast cancer cells. Fig. S11. Silencing of ROCK2 blocks the increased effect of SNORA71A in cell proliferation, migration, invasion and EMT of MDA‐MB‐231 cells. Table S1. Clinical features of breast cancer patients, and the correlationship between SNORA71A expression and different clinical features. Table S2. Primer and siRNA sequences used in this study. Table S3. Statistic analysis ofreads and base from snoRNA sequencing data. Table S4. Mapping statistics of snoRNA from small RNA sequencing data. Table S5. The commonly upregulated and downregulated snoRNA in both two breast cancer cells. Table S6. Mass spectrometry of SNORA71A pull down product. The“Entry name” shows theproteins may bind to SNORA71A. [file MOL2-16-1947-s001.pdf]

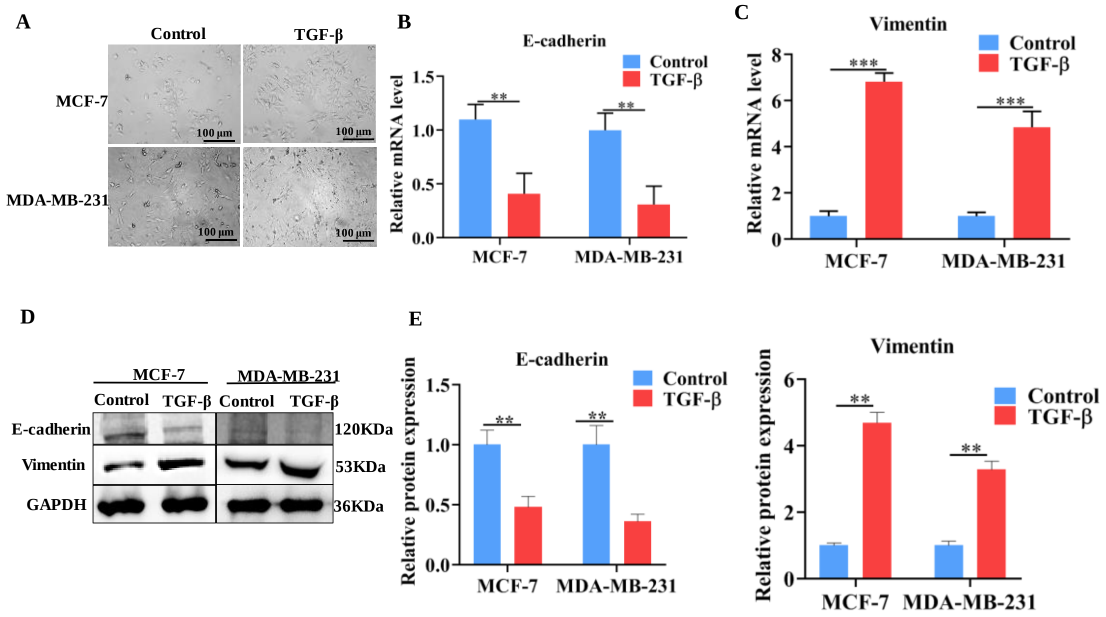

Figure S1 Establishment of EMT model in breast cancer cells. A. Morphology of MCF-7 and MDA-MB-231 cells treated with TGF-β for 48 h. **B.** The mRNA expression of E-cadherin was detected by real-time PCR. **C.** The mRNA expression of Vimentin was detected by real-time PCR. **D.** The protein levels of Vimentin and E-cadherin were detected by western blotting. Each experiment was performed in triplicate. **E.** The protein level in Fig D was analyzed by ImageJ software. Error was defined as SD, t-test, **p<0.01, ***p<0.001.

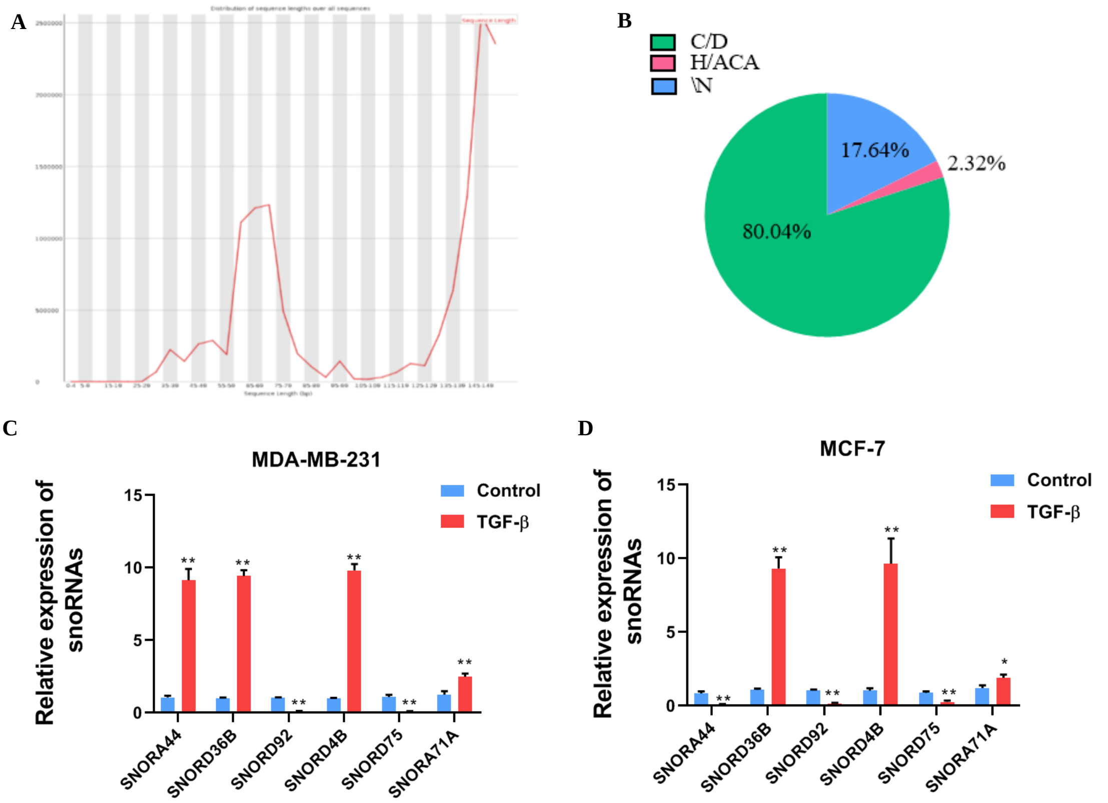

Figure S2 Expression profiles of snoRNA in TGF-β-induced breast cancer cells. snoRNA sequencing was performed in TGF-β-induced MCF-7 and MDA-MB-231 cells and their control cells. **A.** The length distribution of snoRNA. **B.** Classification of snoRNAs in breast cancer cells. **C and D.** Six snoRNAs that differentially expressed in both cell lines were verified by real time PCR. Error was defined as SD, n=3, t-test. *p<0.05, **p<0.01.

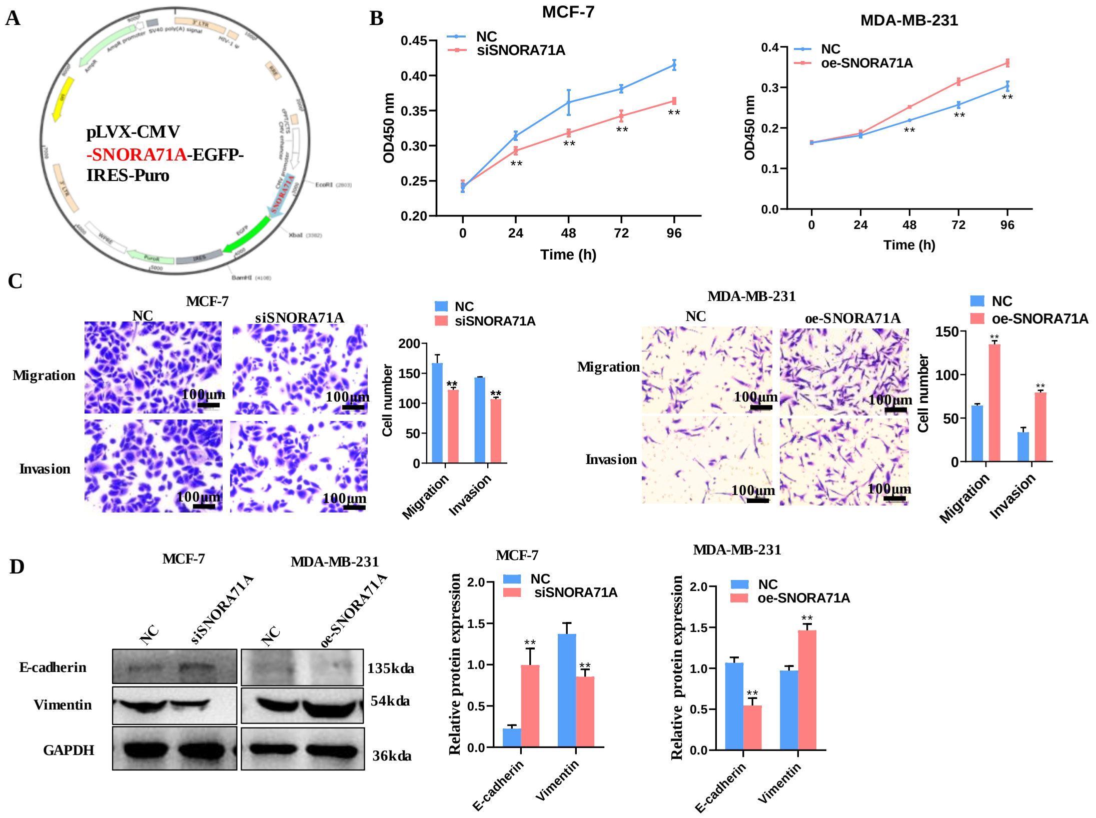

Figure S3 ***SNORA71A*** **promotes cell proliferation, migration, invasion and EMT in breast cancer cells. A.** Overexpression of SNORA71A was obtained by pLVX-CMV-EGFP-IRES-Puro vector using EcoRI(GAATTC)-XbaI(TCTAGA) cloning sites. **B.** The effect of SNORA71A silencing on MCF-7 cell proliferation and SNORA71A overexpression on MDA-MB-231 cell proliferation was performed by CCK-8. NC for silencing was transfected with si-control, NC for SNORA71A overexpression was transfected with empty vector for 48h. n=6, T-test. **p<0.01. **C.** The effect of SNORA71A silencing and overexpression on migration and invasion was performed in MCF-7 and MDA-MB-231 cells by transwell. Scale bar=100μm. n=3, T-test. **p<0.01. **D.** The effect of SNORA71A on the expression of the EMT marker was detected by western blotting after transfection for 48h (three biological replicates). NC for silencing was cells transfected with si-control, NC for SNORA71A overexpression was cells transfected with empty vector. Error was defined as SD, t-test. **p<0.01.

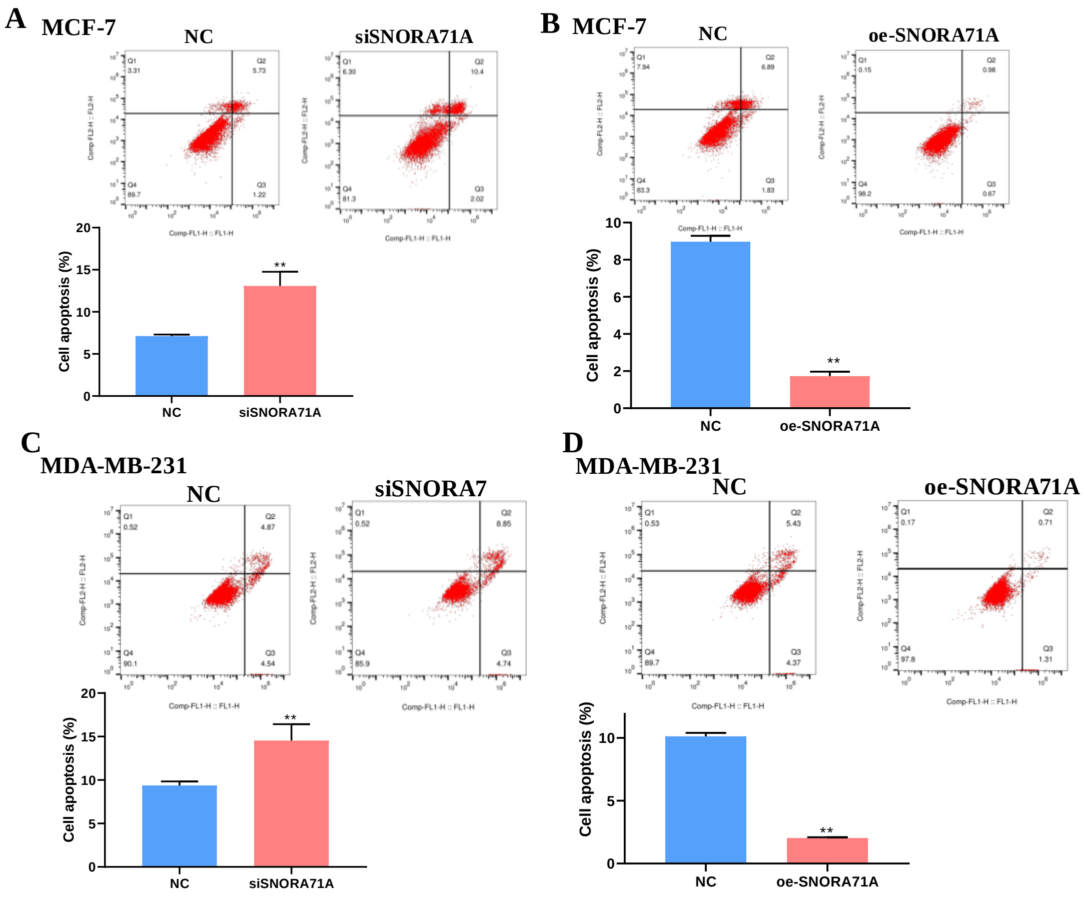

Figure S4 *SNORA71A* inhibits cell apoptosis in breast cancer cells. A-D. Effect of *SNORA71A* on cell apoptosis was performed by flow cytometer. MCF-7 and MDA-MB-231 cells were transfected with si-control, siSNORA71A, empty vector or LVX-*SNORA71A* vector for 48h. NC in figure S4 A and C: cells were transfected with si-control. NC in figure S4 B and D: cells were transfected with empty vector. Error was defined as SD, n=3, t-test, **p<0.01.

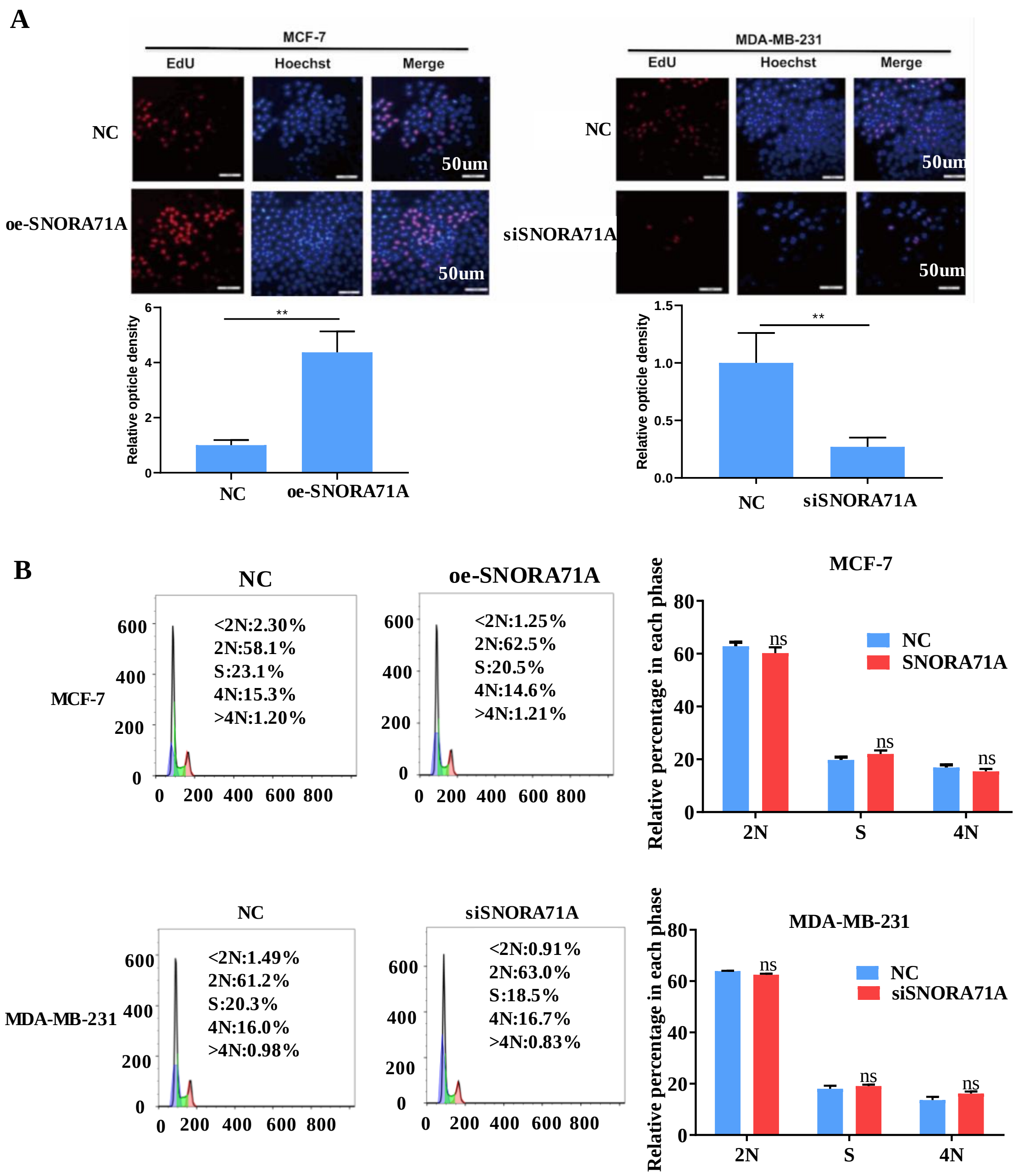

Figure S5 Effect of *SNORA71A* on cell proliferation and cell cycle. A. Cell proliferation was analyzed by immunofluorescence analysis with Edu after transfection for 48h. NC for *SNORA71A* overexpression: cells were transfected with empty vector. NC for *SNORA71A* silencing: cells were transfected with si-control. Scale bar=50μm. n=3, t-test, **p<0.05. **B.** The effect of *SNORA71A* on the cell cycle after transfection for 48h. NC for *SNORA71A* overexpression: cells were transfected with empty vector. NC for *SNORA71A* silencing: cells were transfected with si-control. Error was defined as SD, n=3, t-test, ns: no significant.

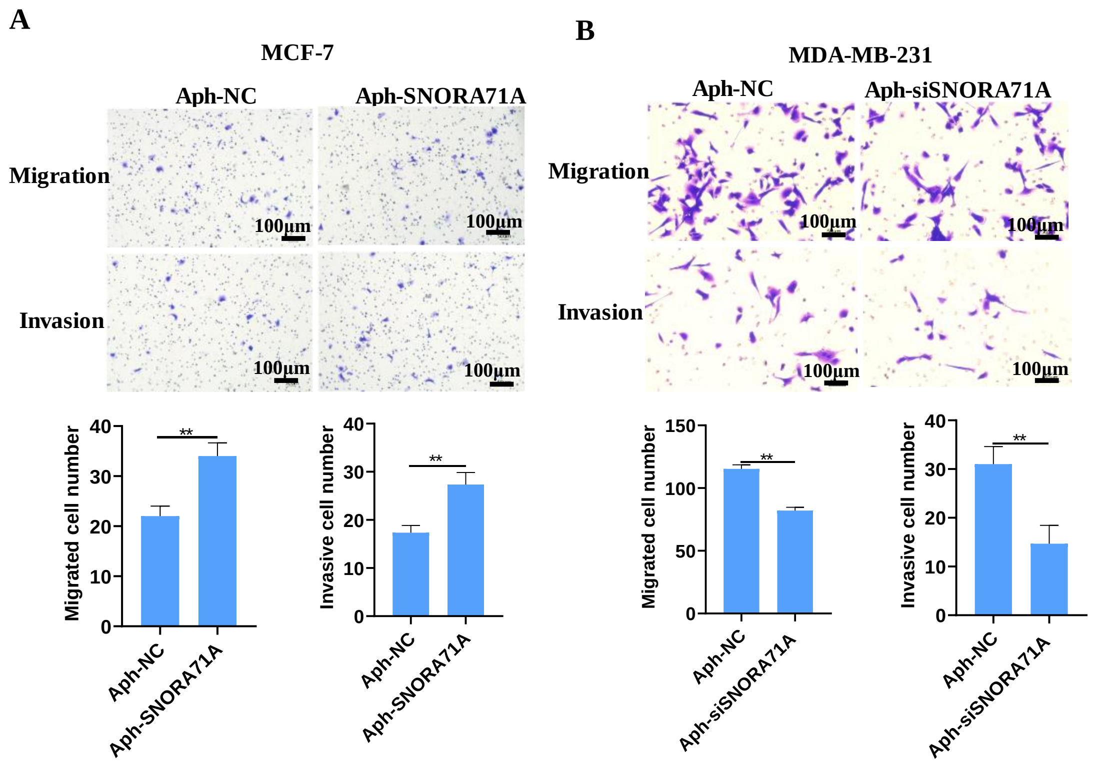

Figure S6 The migration and invasion experiments were also conducted in the presence of aphidicolin (1mg/L). A. The effect of *SNORA71A* overexpression on migration and invasion in the presence of aphidicolin (1mg/L) was detected by transwell assay, in MCF-7cells. Aph-NC: transfected with vector. Scale bar=100μm. **B.** The effect of *SNORA71A* silencing on migration and invasion in the presence of aphidicolin (1mg/L) was detected by transwell assay, in MDA-MB-231 cells. Aph-NC: transfected with si-control. Scale bar=100μm. Error was defined as SD, t-test, **p<0.01.

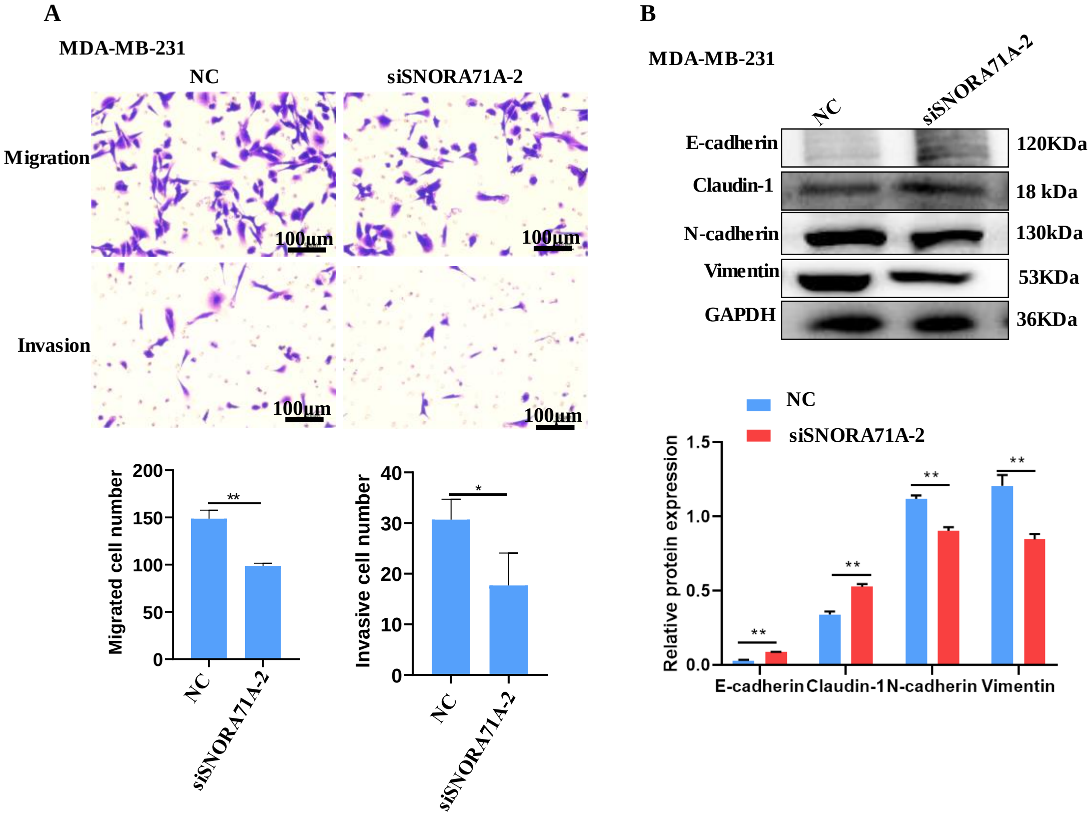

Figure S7 Effect of *SNORA71A* siRNA-2 on migration, invasion and EMT. A. The effect of *SNORA71A* siRNA-2 on migration and invasion was detected by transwell assay in MDA-MB-231 cells. Scale bar=100μm. **B.** The effect of *SNORA71A* siRNA-2 on EMT was detected by transwell assay in MDA-MB-231 cells. NC: cells were transfected with si-control. Error was defined as SD, t-test, *p<0.05, **p<0.01.

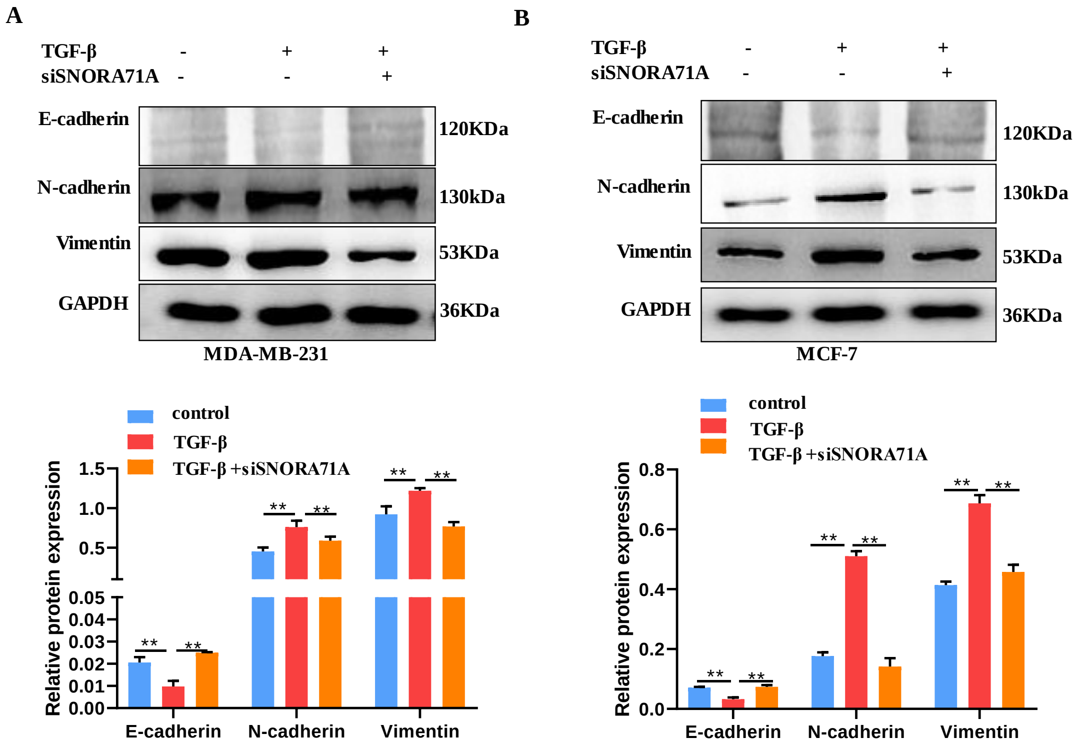

Figure S8 Deficiency of *SNORA71A* abrogated the promote effect of TGF-β on EMT. A. The expression of epithelial marker E-cadherin, and mesenchymal marker N-cadherin and Vimentin in MDA-MB-231 cells was detected by western blot. **B.** The expression of epithelial marker E-cadherin, and mesenchymal marker N-cadherin and Vimentin in MCF-7 cells was detected by western blot. Control: cells without any treatment. TGF-β: cells were only treated with TGF-β for 48 h, and without si-control transfected. TGF-β+siSNORA71A: cells were transfected with siSNORA71A and treated with TGF-β for 48 h. Error was defined as SD, n=3, one-way ANOVA. ** $p<0.01$.

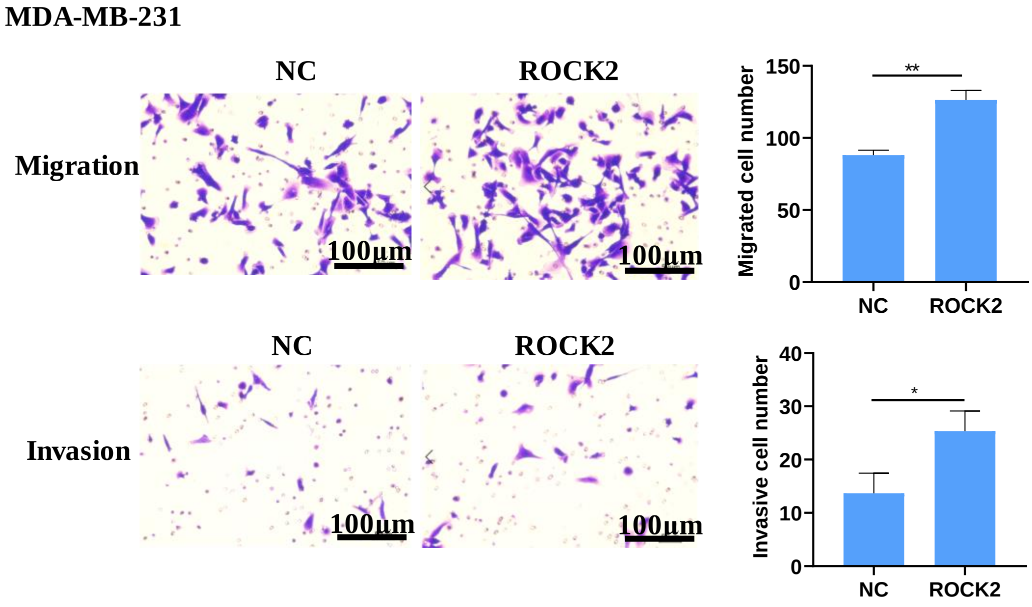

Figure S9 *ROCK2* promotes the migration and invasion of breast cancer cells. The migration and invasion was measured by transwell. NC**:** MDA-MB-231 cells were transfected with empty vector. ROCK2: MDA-MB-231 cells were transfected with ROCK2-overexpressed vector for 48h. Scale bar=100μm. Error was defined as SD, n=3, t-test, *p<0.05, **p<0.01.

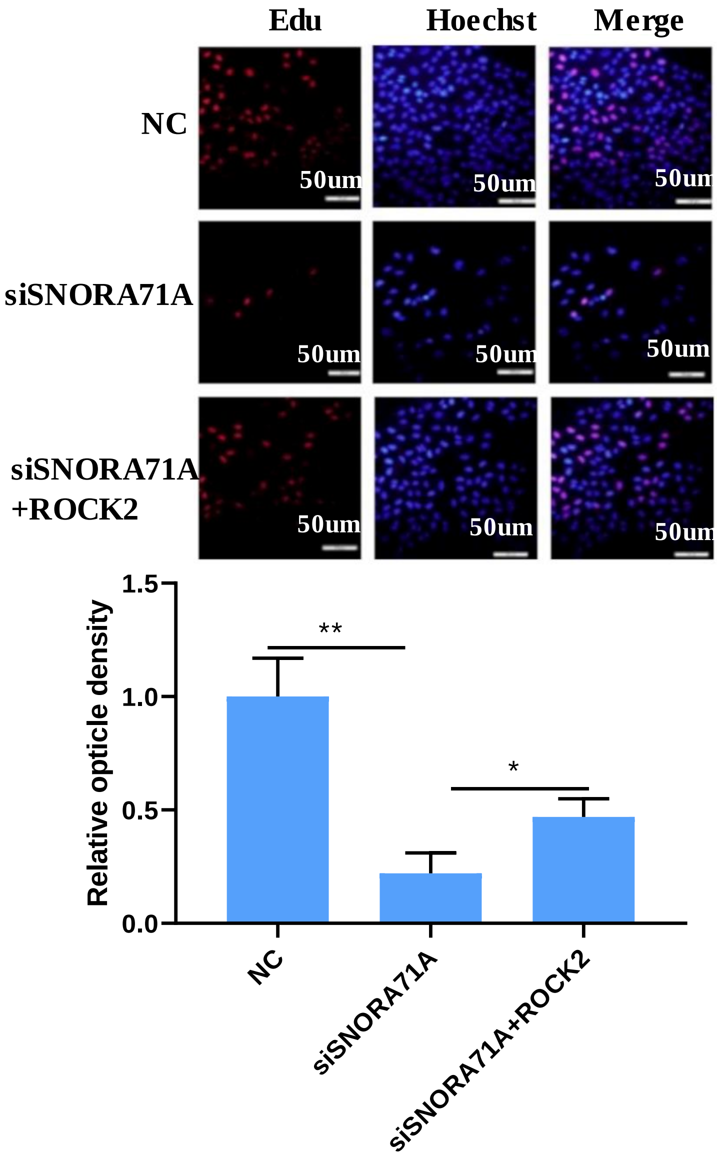

Figure S10 *ROCK2* overexpression significantly blocked the silencing of *SNORA71A* to enhance the proliferation of breast cancer cells. Cell proliferation was analyzed by immunofluorescence analysis with Edu in MDA-MB-231 cells. NC: cells were transfected with si-control. siNORA71A: cells were transfected with siNORA71A. siNORA71A + ROCK2: cells were co-transfected with siNORA71A and *ROCK2*-overexpressed vector for 48h. Scale bar=50μm. Error was defined as SD, n=3, one way-ANOWA, *p<0.05, **p<0.01.

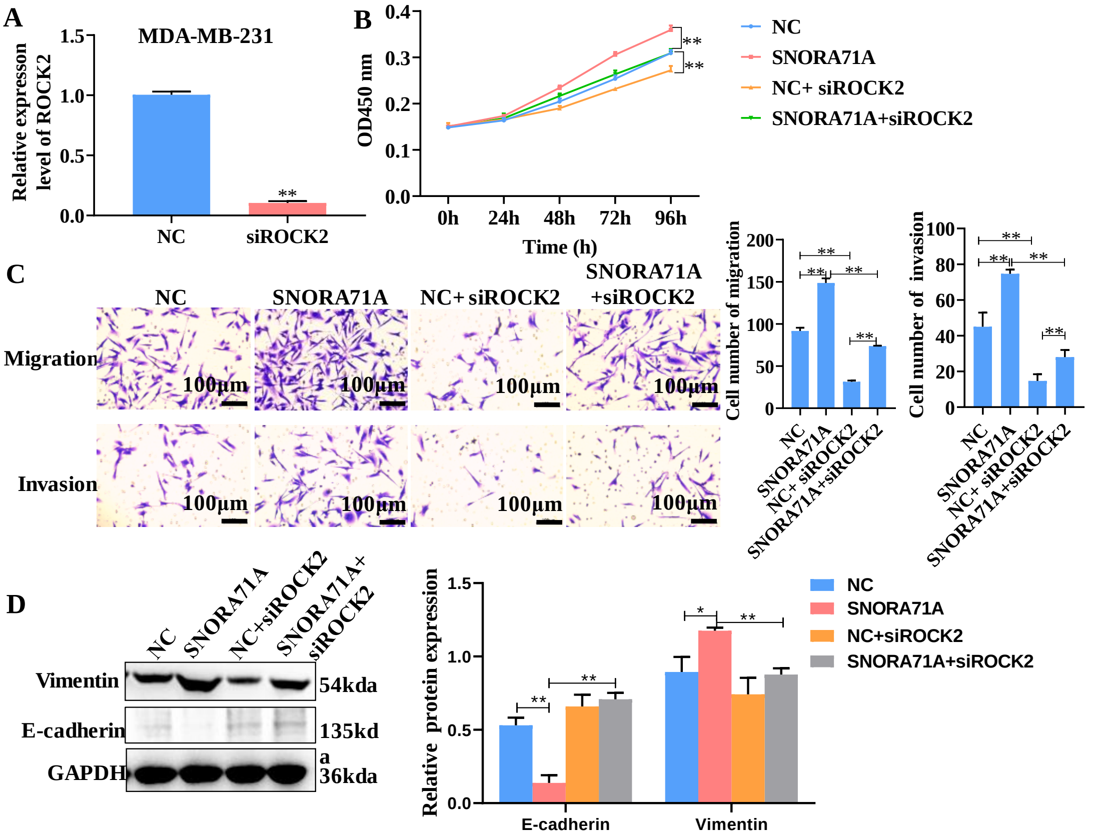

Figure S11 Silencing of *ROCK2* blocks the increased effect of *SNORA71A* in cell proliferation, migration, invasion and EMT of MDA-MB-231 cells. A. Silencing efficiency of *ROCK2* in MDA-MB-231 cells was verified by real time PCR. Cells were transfected for 24h. NC: cells were transfected with si-control. n=3, t-test, **p<0.01. **B.** Cell proliferation was analyzed by CCK-8. MDA-MB-231 cells were transfected with empty vector (NC), *SNORA71A*-overexpressed vector, si-control, or co-transfected with *SNORA71A*-overexpressed vector and siROCK2. n=6, one way-ANOWA, **p<0.01. **C.** Cell migration and invasion were measured by transwell. MDA-MB-231 cells were transfected with empty vector (NC), *SNORA71A*-overexpressed vector, si-control, or co-transfected with *SNORA71A*-overexpressed vector and siROCK2 for 48h. Scale bar=100μm. n=3, one way-ANOWA, **p<0.01. **D.** EMT biomarkers were detected by western blot after transfected for 48h. NC: cells were transfected with empty vector. Error was defined as SD, n=3, one way-ANOWA, *p<0.05, **p<0.01.

Table S1 Clinical features of breast cancer patients, and the correlationship between *SNORA71A* expression and different clinical features.

	Hight	Low	x2	p
Age				
<=60	11	11	**0.832579**	***0.361528***
>60	9	8		
Tumor grade				
High + middle	10	7	**0.255275**	***0.613385***
Low	10	12		
Stage				
I-II	5	14	**7.397644**	***0.006531***
III-IV	15	5		
ER status				
Positive	4	14	**9.242340**	***0.002365***
Negative	16	5		
PR status				
Positive	7	12	**2.067824**	***0.150436***
Negative	13	7		
HER-2 status				
Positive	11	7	**0.665273**	***0.414705***
Negative	9	12		
Lymph node metastasis				
Yes	15	7	**4.322120**	***0.037620***
No	5	12		

Table S2 Primer and siRNA sequences used in this study.

Gene name	Primer sequence
SNORA71A	F: 5’- AGGGAGAGGAACGCTGAAAGAG -3’
	R: 5’- TCGGATGGGATAGGGTGGA -3’
c-myc	F: 5’- CCGCTTCTCTGAAAGGCTCTC -3’
	R: 5’- TCCTCCTCGTCGCAGTAGAAATA -3’
ROCK1	F: 5’- TAACAGAACTAGCTCCAATGCAGAT -3’
	R: 5’- GGTTCTGCACTTCTGCTCCATT -3’
ROCK2	F: 5’- GCAGAAGTGGGTTAGTCGGTTG -3’
	R: 5’- GGCAGTTAGCTAGGTTTGTTTGG -3’
MAPK1	F: 5’- GGAGCAGTATTACGACCCGAGT -3’
	R: 5’- CTTTTCCTTAGGCAAGTCATCCA -3’
SMAD3	F: 5’- GGAGGAGAAATGGTGCGAGAA -3’
	R: 5’- CACAGGCGGCAGTAGATGACA -3’
GAPDH	F: 5’- AGAAGGCTGGGGCTCATT -3’
	R: 5’- TGCTAAGCAGTTGGTGGTG -3’
SNORA71A-siRNA-3	Sense: 5’- CCUGCAUCCGAAAGUGAUCTT -3’
	Antisense: 5’- GAUCACUUUCGGAUGCAGGTT-3’
SNORA71A-siRNA-35	Sense: 5’- GCCUAGGUCAUUGAUAGUGTT -3’
	Antisense: 5’- CACUAUCAAUGACCUAGGCTT -3’
siRNA NC	Sense: 5’-UUCUCCGAACGUGUCACGUTT -3’
	Antisense : 5’-ACGUGACACGUUCGGAGAATT -3’

Table S3 Statistic analysis of reads and base from snoRNA sequencing data.

SampleName	TotalReads_Before	TotalBase_Before	TotalReads_After	ReadsFilter%
MCF-7-TGF-beta	16944326	2.54E+09	14325019	0.845417
MDA-MB-231-TGF-beta	19599682	2.94E+09	15843096	0.808334
MCF-7-blank	15975974	2.4E+09	13228362	0.828016
MDA-MB-231-blank	25097748	3.76E+09	21858160	0.870921

Table S4 Mapping statistics of snoRNA from small RNA sequencing data.

Sample name	Mapped reads	Mapped rate	Unique mapped rate	AllBase	MappedBase
MCF-7-blank	3925210	0.297	0.175	1.46E+09	2.71E+08
MCF-7-TGF-β1	4060399	0.283	0.166	1.62E+09	2.8E+08
MDA-MB-231-blank	6563734	0.3	0.175	2.45E+09	4.57E+08
MDA-MB-231-TGF-β1	4775464	0.301	0.176	1.74E+09	3.33E+08

Table S5 The commonly upregulated and downregulated snoRNA in both two breast cancer cells.

MCF-7 TGF-β-vs- control

snoRNA_id	log2FC	p-value	FDR	Style	MCF-7-TGF-β	MCF-7-control
SCARNA16	-1.17589	0.000518	0.002038	down	24.13556	54.51938
SNORD36B	0.858323	5.89E-06	3.06E-05	up	156.1423	86.11004
SNORD4B	0.712649	1.86E-13	1.91E-12	up	567.9245	346.4783
SNORD92	-0.79624	1.30E-21	2.22E-20	down	454.1426	788.4928
SNORD75	-0.92308	2.83E-24	5.07E-23	down	366.4664	694.7399
SNORA44	0.862438	7.21E-06	3.58E-05	up	151.9555	83.56241
SNORA71A	1.167975	4.61E-27	8.69E-26	up	530.736	236.155

MDA-MB-231 TGF-β-vs-control

snoRNA_id	log2FC	p-value	FDR	Style	MDA-MB-231-TGF--β	MDA-MB-231-control
SCARNA16	0.937449	0.00087	0.003059	up	72.6631	37.93572
SNORD36B	0.710691	6.01E-07	2.96E-06	up	262.5923	160.427
SNORD92	-0.81686	1.92E-26	1.97E-25	down	531.8855	936.8143

SNORD4B	0.73455	1.68E-12	1.17E-11	up	495.6586	297.8488
SNORD75	-0.7591<br>3	6.03E-15	4.94E-14	down	337.9776	571.9306
SNORA44	1.17572<br>5	9.24E-14	6.68E-13	up	251.2845	111.2172
SNORA71A	1.54742	2.09E-52	3.36E-51	up	689.5665	235.8799

Table S6 Mass spectrometry of *SNORA71A* pull down product. The “Entry name” shows the proteins may bind to *SNORA71A*.

Entry name	MW [kDa]	calc. pI	Score Sequest HT: Sequest HT	Abundances
VIME	53.6	5.12	316.02	5982715292
K2C1	66	8.12	86.42	268805169
K2C8	53.7	5.59	120.24	706769169.4
K1C19	44.1	5.14	99.63	807092933.3
K2C7	51.4	5.48	77.68	313299035
K1C9	62	5.24	52.96	127168872
ACTB	41.7	5.48	61.27	755298713
K1C18	48	5.45	72.76	461427116
K1C10	58.8	5.21	67.97	214261895.7
K22E	65.4	8	49.84	82188307.19
ACTS	42	5.39	43.29	1721404.125
K1C14	51.5	5.16	48.47	14553337.81
K2C5	62.3	7.74	33.12	15672340.31
ATPB	56.5	5.4	21.89	13541534.7
HORN	282.2	10.04	17.91	5945953.656
PYC	129.6	6.84	31.43	22774677.31
ATPA	59.7	9.13	23.44	22970567.56
BASP1	22.7	4.63	15.81	10889509.25
K1C16	51.2	5.05	34.91	5539770.875
PLEC	531.5	5.96	24.2	27959468
EF1A1	50.1	9.01	24.25	66832159.75
ALBU	69.3	6.28	14.24	131099441.6
TBB4B	49.8	4.89	15.73	17543150.69
TBB5	49.6	4.89	15.5	4842767.5

DESM	53.5	5.27	23.83	880744.0938
TBA1A	50.1	5.06	10.87	24354244.78
QCR2	48.4	8.63	10.37	9109867.766
HLAA	40.9	6.99	6.06	1741575.359
PDIA3	56.7	6.35	6.6	4496622.828
K1C15	49.2	4.77	21.35	18354096
KRT84	64.8	7.56	12.3	66234055.5
K1C13	49.6	4.96	15.92	
ECHB	51.3	9.41	15.28	7279035.5
DCD	11.3	6.54	7.72	6340932.813
K2C80	50.5	5.67	7.55	3999653.813
DHE3	61.4	7.8	4.03	1608998.563
SQOR	49.9	9.11	4.92	1109074.063
RINI	49.9	4.82	5.28	4206645.75
SAM50	51.9	6.9	8.28	4291151.031
HNRH1	49.2	6.3	2.73	1399073.125
ODO2	48.7	8.95	5.88	2410648.75
EF1G	50.1	6.67	3.9	1323564.328
FLOT1	47.3	7.49	2.16	2018860.578
CN37	47.5	9.07	2.53	317828.2188
ENOA	47.1	7.39	4.65	2220025.563
HS2ST	41.9	8.69	4.11	1736456.594
IF2G	51.1	8.4	2.54	749692.9688
AATM	47.5	9.01	5.52	1078920.344
FILA2	247.9	8.31	3.07	716156.3125
PCCB	58.2	7.64	4.23	2480666.031
HLAB	40.5	6.54	2.24	699673.25
H4	11.4	11.36	5.3	1947346.125
OST48	50.8	6.55	2.76	1639184.375

PRPC	17	4.84	0	617496.8125
EIF3F	37.5	5.45	2.21	122225.0234
GLYM	56	8.53	2.12	1636909.375
YBOX1	35.9	9.88	3.49	674846.125
EFTU	49.5	7.61	3	2014765.5
NEST	177.3	4.36	1.82	933833.3125
HSP77	40.2	7.87	1.74	315987.625
RL3	46.1	10.18	1.8	3274449.5
ARP2	44.7	6.74	1.72	413454.3125
PAIRB	44.9	8.65	2.78	637975.3125
G3BP2	54.1	5.55	2.14	413944.9688
RL4	47.7	11.06	3.01	1449814.156
CAVN1	43.5	5.6	0	904367.9688
AP2M1	49.6	9.54	4.15	3008815.625
SPTC1	52.7	6.01	2.16	326190.1563
MCCB	61.3	7.68	3.94	784114.9688
CATD	44.5	6.54	3.94	2756145.781
ANT3	52.6	6.71	2.24	3205462.75
H13	22.3	11.02	1.93	667675.25
QCR1	52.6	6.37	2.44	1815183.094
CALR	48.1	4.44	4.52	1248505.813
PDIA6	48.1	5.08	2.01	404873.7813
CD63	25.6	7.81	2.06	653748.8125
THIM	41.9	8.09	0	592281
DEF1	10.2	6.99	4.39	2433412.25
APMAP	46.5	6.16	0	469389.5625
SCPDL	47.1	9.14	3.63	722909.9531
CCD47	55.8	4.87	0	157328.6875
TXND5	47.6	5.97	2.07	766384.5

ACACA	265.4	6.37	0	
FUMH	54.6	8.76	1.75	
KRT85	55.8	6.55	1.82	14865685
HBE	16.2	8.63	0	195763.3594
HBA	15.2	8.68	0	211352.125
DSG1	113.7	5.03	0	
SERPH	46.4	8.69	2.02	566357.375
IDHP	50.9	8.69	1.96	
G3BP1	52.1	5.52	0	283118.4063
PGK2	44.8	8.54	2.5	455412.9688
BGAL	76	6.57	1.82	
PREB	45.4	7.88	0	437904.6875
STAB1	275.3	6.49	0	
SOAT1	64.7	8.94	0	243542.2031
IF4A1	46.1	5.48	0	307952.4063
SEMG1	52.1	9.29	0	634646.6875
RL40	14.7	9.83	2.23	1680125.125
CISY	51.7	8.32	1.86	1158121.625
CP1B1	60.8	8.98	0	96157.98438
GNAS1	111	5.03	0	321568.8438
IGHA1	37.6	6.51	2.03	
DHC24	60.1	8.16	0	301191.75
AL1A3	56.1	7.25	1.82	368203.7813
APOA1	30.8	5.76	0	293616.3438
DLDH	54.1	7.85	1.91	597813.625
DX39A	49.1	5.68	0	145334.2344
TRFL	78.1	8.12	0	119599.6563
RPGF2	167.3	6.67	0	267367.3125
H2A2C	14	10.9	0	628391.25

SCMC1	53.3	6.33	2.2	821112.9375
S10A7	11.5	6.77	1.71	
PRS8	45.6	7.55	0	
LAP2B	50.6	9.38	0	174039.8125
SYNE2	795.9	5.36	0	
GGNB2	79	6.38	0	846007.9375
SYDC	57.1	6.55	0	103416.9688
AGK	47.1	8.09	0	183342.0781
SF3B1	145.7	7.09	0	
PCAT1	59.1	6.02	0	
NOE2	51.4	7.94	0	
KPRP	64.1	8.27	0	435403.2813
